# Predicting the onset of mental health problems in adolescents

**DOI:** 10.1017/S003329172500087X

**Published:** 2025-04-30

**Authors:** Jiangyun Hou, Laurens Mortel, Arne Popma, Dirk Smit, Guido van Wingen

**Affiliations:** 1 Department of Psychiatry, University of Amsterdam, Amsterdam, The Netherlands; 2 Amsterdam Neuroscience, Amsterdam, The Netherlands; 3 Amsterdam Public Health, Amsterdam, The Netherlands

**Keywords:** mental health problems, CBCL, adolescents, machine learning, prediction

## Abstract

**Objective:**

Mental health problems are the major cause of disability among adolescents. Personalized prevention may help to mitigate the development of mental health problems, but no tools are available to identify individuals at risk before they require mental health care.

**Methods:**

We identified children without mental health problems at baseline but with six different clinically relevant problems at 1- or 2-year follow-up in the Adolescent Brain Cognitive Development (ABCD) study. We used machine learning analysis to predict the development of these mental health problems with the use of demographic, symptom and neuroimaging data in a discovery (N = 3236) and validation (N = 3851) sample. The discovery sample (N = 168–513 per group) consisted of participants with MRI data and were matched with healthy controls on age, sex, IQ, and parental education level. The validation sample (N = 84–231) consisted of participants without MRI data.

**Results:**

Subclinical symptoms at 9–10 years of age could accurately predict the development of six different mental health problems before the age of 12 in the discovery and validation sample (AUCs = 0.71–0.90). The additive value of neuroimaging in the discovery sample was limited. Multiclass prediction of the six groups showed considerable misclassification, but subclinical symptoms could accurately differentiate between the development of externalizing and internalizing problems (AUC = 0.79).

**Conclusions:**

These results suggest that machine learning models can predict conversion to mental health problems during a critical period in childhood using subclinical symptoms. These models enable the personalization of preventative interventions for children at increased risk, which may reduce the incidence of mental health problems.

## Introduction

Mental health problems are the major cause of disability among adolescents (Armocida et al., 2022). They are characterized by emotional or behavioral disturbances that significantly affect a child’s ability to learn and function at school, home, or social settings. It is estimated that 17% of adolescents are suffering from mental disorders (Castelpietra et al., 2022). The amount of adolescents with mental disorders has only been increasing over the past years and is one of the main health challenges for society (Lebrun-Harris, Ghandour, Kogan, & Warren, 2022). An important objective to improve health outcomes is to prevent the development of disease. However, preventive psychiatry has had little impact so far as preventive programs only have small effects (Fusar‐Poli et al., 2021; Werner-Seidler et al., 2021). This calls for the personalization of preventative interventions which requires the identification of individuals that are at risk for the development of these disorders.

Meta-analyses have identified multiple risk factors for the development of mental health problems. Those factors each only confer a small risk at the population level and can therefore not predict the development of individuals (Kim et al., 2020; Pedersen et al., 2023). Machine learning has been increasingly used to develop predictive models for a variety of mental disorders. By combining multiple risk factors in a multivariate manner, machine learning algorithms can identify patterns that can be used to predict the likelihood of developing that disorder in individuals. For example, demographic data can be used to predict the development of attention-deficit/hyperactivity disorder (ADHD) (Garcia-Argibay et al., 2023), while personality traits and imaging data can predict the development of anxiety disorders (Chavanne et al., 2022). However the majority of studies are limited to one particular disorder, have been conducted in at-risk samples, and/or have only modest predictive capacity (Chan et al., 2023; Senior, Fanshawe, Fazel, & Fazel, 2021; Whelan et al., 2014). The factors underlying the development of different mental health problems therefore remain unclear. Such information could enable personalized prevention strategies to mitigate the mental health burden among youth.

For the current study, we used data from the largest longitudinal neuroimaging study in youth, the Adolescent Brain Cognitive Development (ABCD) study (Jernigan, Brown, & Dowling, 2018). We selected individuals who were healthy at baseline and developed various clinically relevant mental health problems during the 2-year follow-up period. The identification of individuals was based on parental report of emotional and behavioral problems using a standard questionnaire (Child Behavior Checklist [CBCL 6–18]) to identify six different categories of disorders in accordance with the diagnostic and statistical manual of mental disorders 5 (DSM-5): ADHD problems, anxiety problems, conduct problems, depressive problems, oppositional defiant problems, and somatic problems (Achenbach, 2013). We extracted demographic, symptom, and neuroimaging data at baseline and applied machine learning methods to (1) predict the development of each of these mental health problems on the individual level at follow-up using unimodal and multimodal data, and (2) identify the most informative variables in the prediction models.

## Methods

### Participants

We selected participants from the ABCD study using DSM-oriented scales from the CBCL, defining individuals with a t-score <65 at baseline and every follow-up as controls, while those with a t-score <65 at baseline and ≥65 at 1- or 2-year follow-up were categorized into clinical groups. The CBCL is constructed to identify abnormal behavior and has a one-tailed distribution, where 65 is the lenient criterion to identify abnormal behavior that has a better balance between sensitivity and specificity than the strict 70 criterion when compared with clinical interview (Krol, De Bruyn, Coolen, & van Aarle, 2006). We identified 454 individuals with ADHD problems, 652 with anxiety problems, 403 with conduct disorder problems, 757 with depressive problems, 434 with oppositional defiant problems, 983 with somatic problems, and 4842 controls. For the first cohort, we only selected participants with usable MRI data, and excluded participants with a psychiatric history at baseline (defined as having ever received mental health or substance abuse services), resulting in 170 participants with ADHD problems, 281 with anxiety problems, 164 with conduct problems, 336 with depressive problems, 171 with oppositional defiant problems, 521 with somatic problems, and 3004 controls (Table 1). Finally, we matched groups on age, sex, IQ, and education level of their parents to obtain six matched and balanced control groups (168 ADHD vs. 168 controls, 275 anxiety problems vs. 275 controls, 163 conduct problems vs. 163 controls, 331 depressive problems vs. 331 controls, 168 oppositional defiant problems vs. 168 controls, 513 somatic problems vs. 513 controls). When we built the models, we replaced missing demographic and symptom values using the mean of these features for train set and test set separately to avoid data leakage. Missing MRI data were not imputed as these data generally missing on the whole (i.e. MRI was not measured). Therefore, we included participants without usable MRI data in a second cohort as a second independent test set for models that did not include MRI. Data can be obtained through registration with the ABCD study at https://nda.nih.gov/abcd.

**Table 1.** Demographic data of the first cohort of included adolescents. Cases are participants without clinically relevant symptoms at baseline (CBCL t-score <65) but with clinically relevant symptoms at 1- or 2-year follow-up (CBCL t-score ≥65). Matched controls did not have clinically relevant symptoms at baseline nor at follow-up (CBCL t-score <65). The race of ABCD participants is reported by https://abcdstudy.org/scientists/data-sharing/baseline-data-demographics-2-0/.

	ADHD		Anxiety problems		Conduct problems	
	Cases	Controls	Cases	Controls	Cases	Controls
Sample size	168	168	275	275	163	163
Age (mean ± SD)	9.91 ± 0.65	10.02 ± 0.66	9.92 ± 0.63	9.96 ± 0.66	9.87 ± 0.63	9.95 ± 0.62
Gender male (%)	48.81%	48.81%	50.91%	50.91%	44.17%	44.17%
IQ (mean ± SD)	96.62 ± 16.04	96.55 ± 16.10	102.76 ± 16.81	102.79 ± 16.81	96.76 ± 15.59	96.76 ± 15.58
EA (mean ± SD)	16.68 ± 2.45	16.79 ± 2.41	17.06 ± 2.48	17.05 ± 2.43	16.10 ± 2.95	16.56 ± 2.64

	Depressive problems		Oppositional Defiant problems		Somatic problems	
	Cases	Controls	Cases	Controls	Cases	Controls
Sample size	331	331	168	168	513	513
Age (mean ± SD)	9.92 ± 0.62	9.99 ± 0.64	9.94 ± 0.65	10.05 ± 0.63	9.86 ± 0.62	9.99 ± 0.64
Gender male (%)	46.83%	46.83%	57.14%	57.14%	54.78%	54.78%
IQ (mean ± SD)	102.66 ± 15.88	102.75 ± 15.94	99.57 ± 16.58	99.64 ± 16.52	101.13 ± 14.88	101.17 ± 14.87
EA (mean ± SD)	16.85 ± 2.59	17.07 ± 2.52	16.73 ± 2.66	17.01 ± 2.37	16.72 ± 2.47	17.00 ± 2.40

### Features

#### Clinical data

The Child Behavior Checklist for Ages 6–18 (CBCL/6–18), reported by parents, is related to the behavioral and emotional problems of school-aged children. It includes 119 items, such as ‘argues a lot’, ‘cannot sit still, restless, or hyperactive’, ‘too fearful or anxious’, to describes the child now or within the past 6 months, rated along the 0-to-2 scale: 0 = not true; 1 = somewhat/sometimes true; 2 = very true/often true. The scale of each item is the raw score. In line with recent research, we used raw scores rather than age and sex-adjusted t-scores (Barch et al., 2021).

We included predictors from previous publications besides demographics and background of DSM-5 (Supplementary Table S14). For ADHD, neurocognition, handedness, screen time, parental monitoring, school involvement, and socioeconomic disadvantage contributed meaningfully to prediction (van Lieshout et al., 2017; Weigard et al., 2022). Irregularities in sleeping schedules and family environment in childhood were associated with depression and anxiety disorders (Ho et al., 2022; Ong, Wickramaratne, Tang, & Weissman, 2006; Wang, Tian, Guo, & Huebner, 2020). Besides, family history and sleep quality were considered important risk factors for depression symptoms (Ho et al., 2022). Family environment and history were associated with oppositional defiant disorder (Mohammadi et al., 2019). We used these as our demographic and symptom features for all included disorders.

### Imaging data

The ABCD imaging data included structural MRI, diffusion MRI, and functional MRI data. For specific imaging-derived measures, please see Supplementary Table S22. We used the following imaging-derived measures: *sMRI*: regional volume, cortical thickness, cortical area, sulcal depth in subcortical and cortical ROIs; *rsfMRI*: correlation within and between cortical networks, correlation between cortical networks and subcortical ROIs, temporal variance in subcortical and cortical ROIs, and in regions defined by the Gordon atlas; *dMRI*: Diffusion Tensor Imaging (DTI) analysis: fractional anisotropy, mean diffusivity longitudinal (or axial) diffusivity and transverse (or radial) diffusivity, fiber tract volume, within DTI atlas tract, within subcortical ROI, with cortical ROI with full shell and with inner shell; *Restriction Spectrum Imaging*: restricted normalized isotropic, restricted normalized directional, restricted normalized total and free normalized isotropic diffusion in AtlasTrack fiber segmentation, aseg subcortical segmentation, peri-cortical white matter Desikan cortical parcellation and peri-cortical white matter Destrieux cortical parcellation; *task fMRI*: beta weight and standard error of the mean in subcortical and cortical ROIs and in cortical ROIs for run1 and run2, and average Beta weight and Standard error of the mean for monetary incentive delay (MID), stop signal task (SST), emotional n-back (EN-back) task fMRI. The ABCD image processing pipeline uses a combination of automated and manual approaches, including general and modality-specific corrections to address known challenges such as head motion, distortion, and intensity inhomogeneities. For a comprehensive description, please see Hagler et al. (2019).

### Analysis

**Figure 1.**
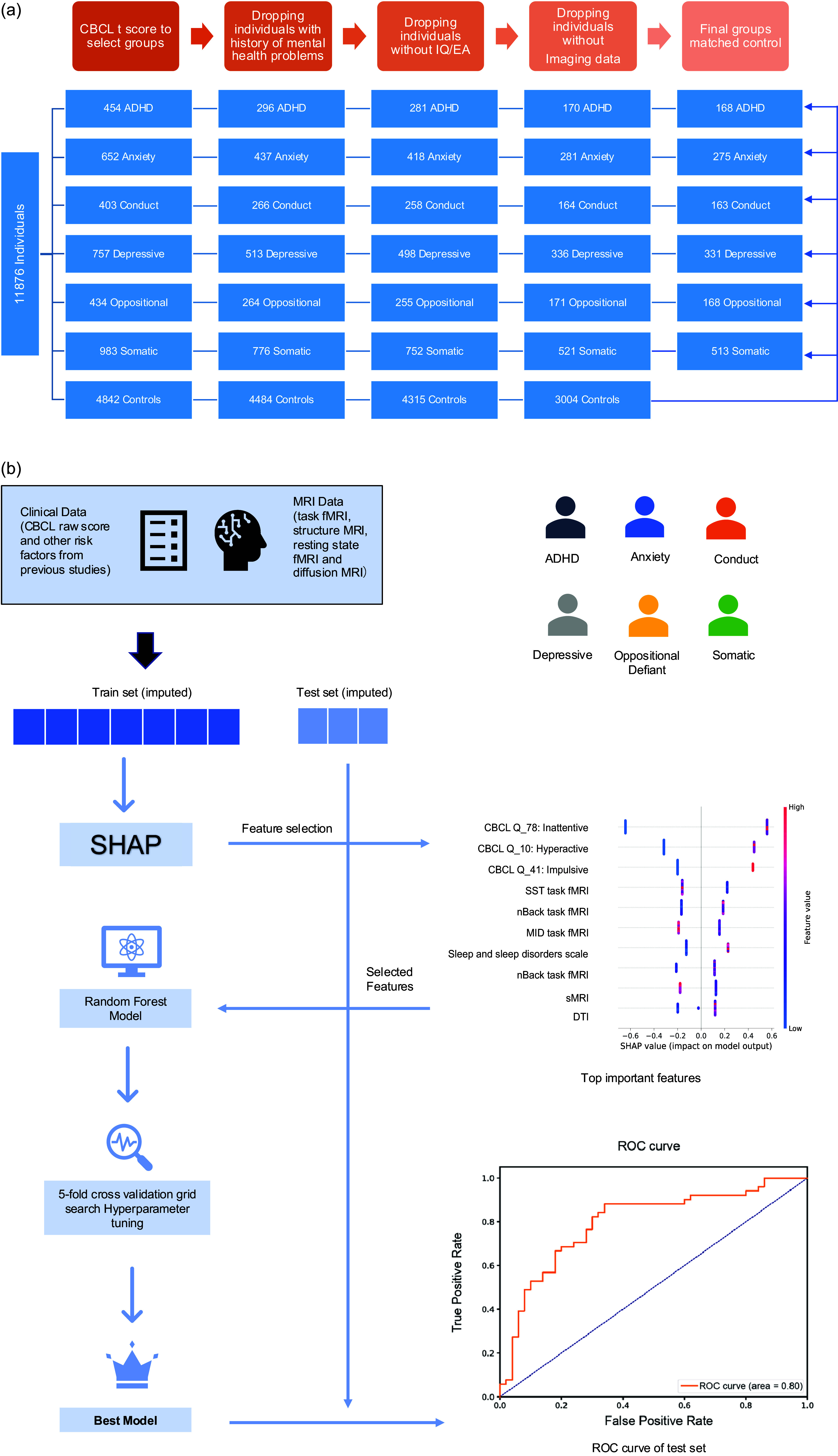
(a) Flowchart for the selection of research participants from the ABCD study. The steps used to select the study sample are shown; (b) Flowchart of analysis pipeline. Participants with different clinically relevant problems and their controls were selected from the ABCD study; samples were split into training and test sets; features were selected using SHAP; and random forest classifiers were trained using 5-fold cross-validation. Performance was assessed on the test sets using the area under the receiver operating characteristic (ROC) curve, which plots the true positive rate against the false positive rate at all classification thresholds.

We used a random forest classifier to predict the development of these six different psychiatric disorders. The first cohort consisted of participants for whom neuroimaging data were available. We used 70% of the total data for training (i.e. tuning hyperparameter and validation) and the remaining 30% for testing (first test set). This train/test ratio ensured sufficient data for testing with the number of participants per group, as test variability is large for small samples (Flint et al., 2021). The second cohort consisted of participants for whom no neuroimaging data were available, who were all included in a second test set based on a model that did not include imaging features (N: ADHD = 110, anxiety problems = 138, conduct problems = 92, depressive problems = 160, oppositional defiant problems = 84, somatic problems = 231, and control = 3036). As the total number of features was 52482 (Supplementary Table S22), we used SHAP (SHapley Additive exPlanation) for feature selection. SHAP was used as a substitute for feature importance permutation to alleviate the computational workload and was used to explain the results and analyze feature importance for each model. For this procedure, we used SHAP to obtain the most informative features from XGBoost tree-based models and then included these features into the random forest model as described. We utilized the Scikit-learn package in Python and used a 5-fold cross-validation grid search to select the best parameters and the area under the receiver operating characteristic curve (AUC) as the performance measure of this model on test sets. Performance was evaluated using AUC on the test set (Figure 1). AUC between 0.7 and 0.8 was considered acceptable, AUC between 0.8 and 0.9 as excellent, and AUC > 0.9 as outstanding (Hosmer, Lemeshow, & Sturdivant, 2013).

To assess whether the results were algorithm-dependent, we additionally used three other commonly used machine learning algorithms: Logistic Regression, Support Vector Machine (SVM) with an RBF kernel, and Multi-Layer Perceptron (MLP). Each model was evaluated on important features from SHAP using 5-fold stratified cross-validation and the cross-validated AUC and standard deviation were computed for each model to assess both performance and stability.

## Results

In this study, we identified seven different groups (ADHD problems, anxiety problems, conduct problems, depressive problems, oppositional defiant problems, somatic problems, and controls) and matched each symptom group to controls based on age, sex, IQ, and education level of parents to obtain six matched and balanced samples (N per group = 163–513). Sample characteristics are presented in Table 1

## Multimodal prediction

**Figure 2.**
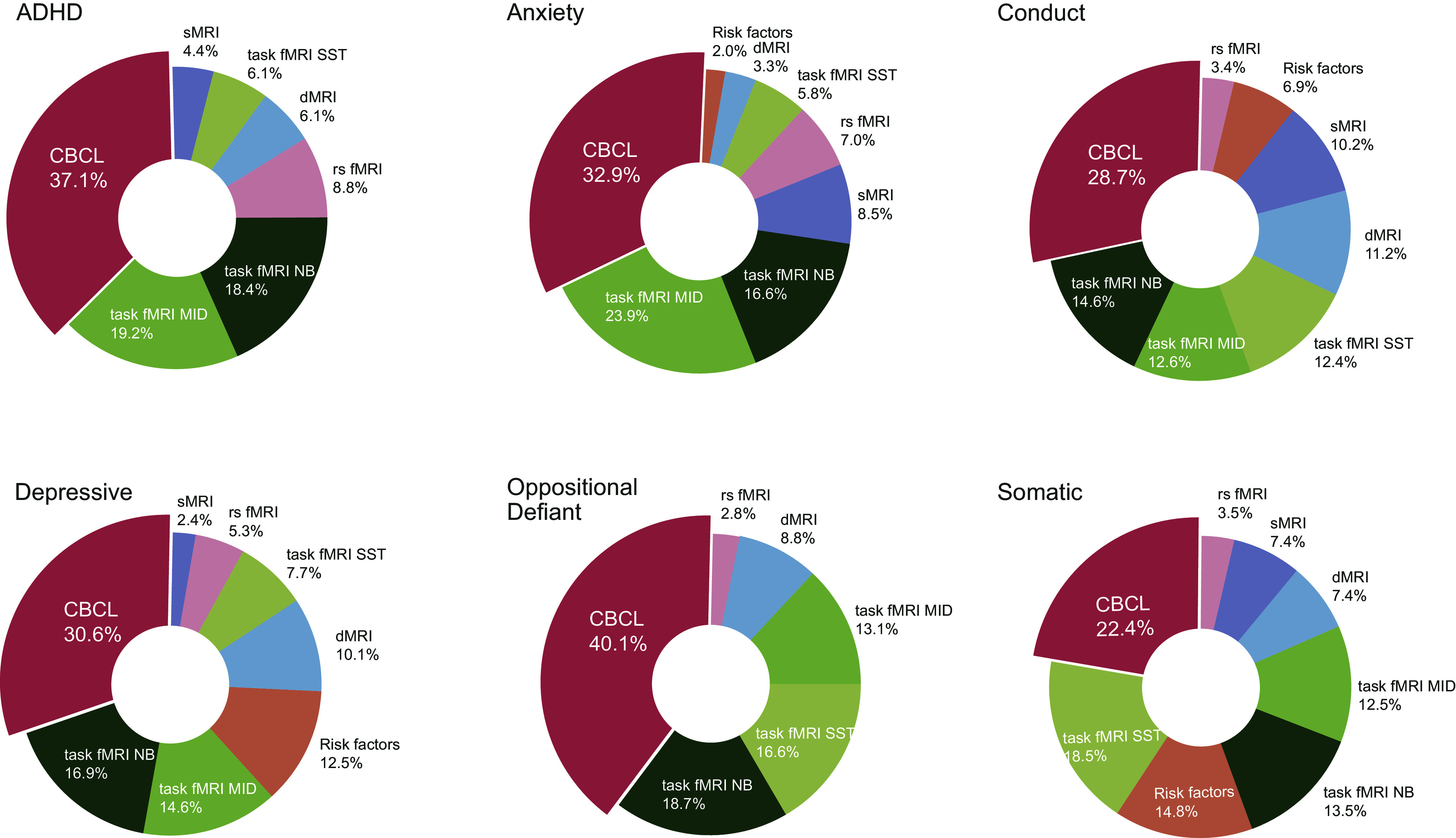
Pie charts illustrating the relative contribution of different feature modalities to the multimodal prediction of the development of clinically relevant mental health problems in adolescents.

We used the risk factors, symptoms, and neuroimaging data at baseline as features for our machine learning models to determine whether multimodal data are suitable for predicting later mental health problem status on an individual level and which features are most important for this prediction. We used 70% of the dataset for model training and 30% to test model performance. Due to the large feature set, we used feature selection with SHAP feature importance on the training set. Random forest classification revealed excellent discrimination for most predictive models (Hosmer et al., 2013) (AUC: ADHD: AUC = 0.80, anxiety problems: AUC = 0.85, conduct: AUC = 0.82, depressive problems: AUC = 0.83, oppositional defiant problems: AUC = 0.82, somatic problems: AUC = 0.69) (Supplementary Figure S2). Inspection of the most important features (Figure 2; Supplementary Figure S1; Supplementary Tables S1–S6) suggests that different CBCL items were highly influential and were supported by various neuroimaging measures.

To assess whether the performance of other machie learning classifiers was comparable, we additionally evaluated the performance of Logistic Regression, SVM, and MLP models across the six mental health problems. Across all problems, Logistic Regression achieved test AUCs ranging from 0.70 (somatic problems) to 0.84 (ADHD); MLP showed test AUCs ranging from 0.62 (somatic problems) to 0.78 (depressive problems), excepted conduct problems (test AUC = 0.54). In contrast, SVM exhibited relatively lower performance, particularly for anxiety problems (test AUC = 0.51), conduct problems (test AUC = 0.47), and somatic problems (test AUC = 0.52) (Supplementary Table S20).

## Unimodal prediction

**Figure 3.**
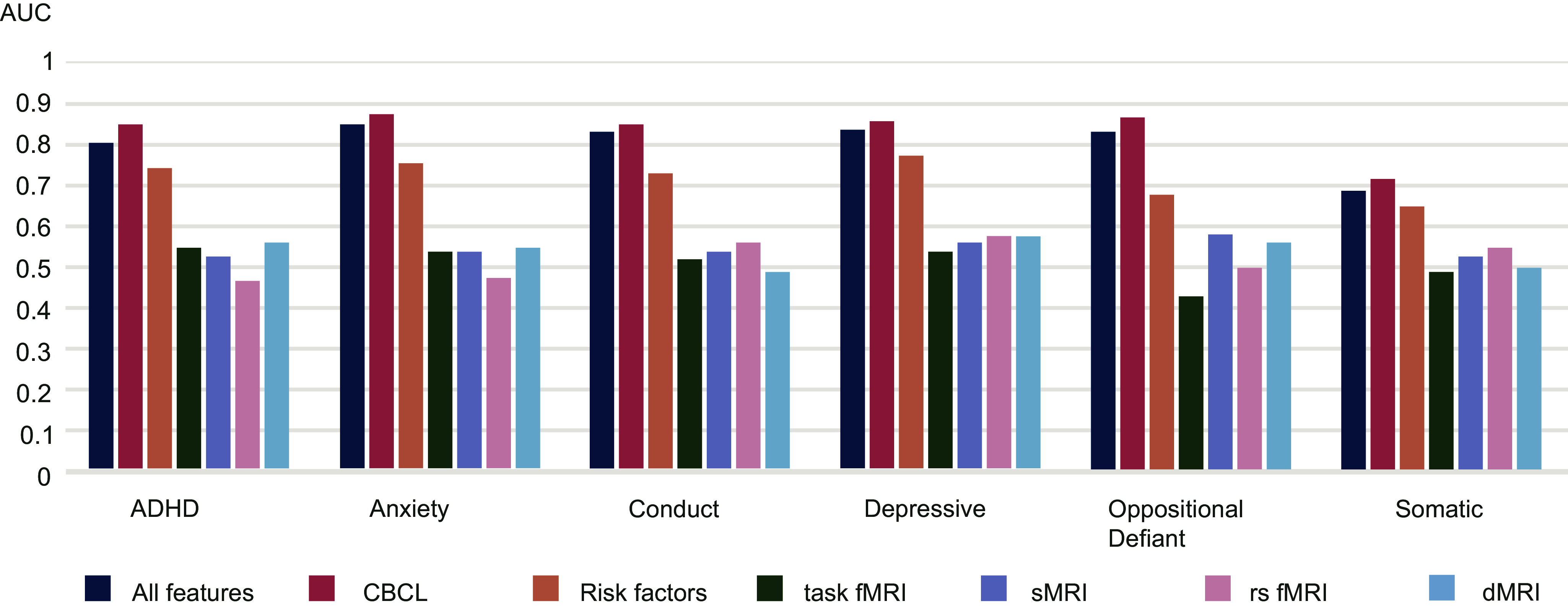
Performance (AUC) of the models predicting the development of clinically relevant mental health problems in adolescents for each of the multimodal and unimodal predictions.

To disentangle the influence from CBCL and other features on the prediction, we built another six models with only CBCL features, task-fMRI, structural MRI, resting state-fMRI, diffusion MRI, and other known risk factors reported in the literature for every mental health problem group. The AUCs for all models are shown in Figure 3, and the ROC curves using the CBCL in the first test set are shown in Supplementary Figure S3. This figure illustrates that CBCL items were the most important features for predicting the mental health problems of interest, and generally performed better without the addition of other features. As we had excluded participants who did not have neuroimaging features, we tested the CBCL model on a second sample of participants who were excluded in the initial analysis. This resulted in better performance: ADHD: AUC = 0.90, anxiety problems: AUC = 0.83, conduct problems: AUC = 0.78, depressive problems: AUC = 0.83, oppositional defiant problems: AUC = 0.89, and somatic problems: AUC = 0.79 (Supplementary Figure S4).

To evaluate whether a simple summation of CBCL items could be just as informative as the use of all individual items, we computed CBCL total scores of the most important features (Supplementary Table S7) from the SHAP of every model in the first dataset for the first test set and second test set. ROC curves of the test data showed that performance was as good as using all individual items (Supplementary Figures S5–S6). For the first test set, inspection of the sensitivity/specificity tradeoffs suggests that a summary score of only 3–4 results in optimal prediction accuracy, with somatic problems (67%) scoring considerably lower than other mental health problems (80%–84%; Supplementary Tables S8–S13). When avoiding misclassification of youth that do not develop clinically relevant mental health problems, specificity can be increased to >90% with a summary score of 4–8, which reduces sensitivity to 37%–75%. For the second test set, the optimal prediction accuracy decreased to 69%–83% with a summary score of only 3–5 (Supplementary Tables S14–S19). Specificity can be increased to >90% with a summary score of 5–8 which reduces sensitivity to 41%–71%.

To evaluate whether the CBCL items that predict later problems resembled those that are used for diagnosis, we assessed the overlap between items for CBCL total scores and items for the original identification of problems (Supplementary Table S7). Venn diagrams indicate that there was little overlap between the CBCL items for diagnosis and prognosis, which was confirmed by low Jaccard indices (0.07–0.23; Supplementary Figure S7).

The accuracy of the other algorithms using CBCL items only showed comparable performance, with Logistic Regression achieved test AUCs ranging from 0.702 (Somatic Problems) to 0.882 (Anxiety Problems); SVM with test AUCs ranging from 0.692 (Somatic Problems) to 0.891 (Anxiety Problems); MLP with test AUCs ranging from 0.666 (Somatic Problems) to 0.852 (Conduct Problems) (Supplementary Table S21).

## Multiclass classification

**Figure 4.**
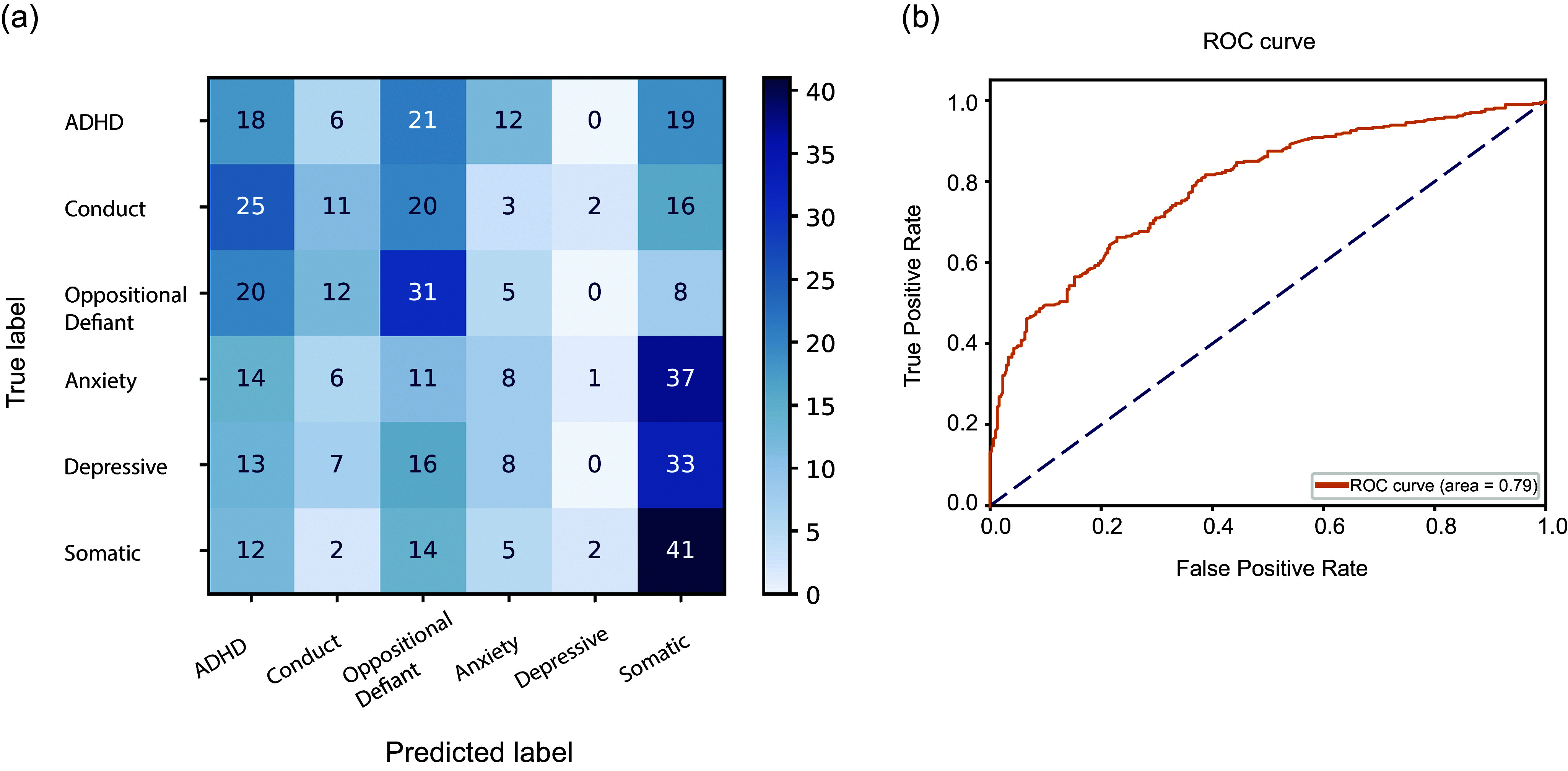
(a) The confusion matrix for the multiclass classification using CBCL data, illustrating misclassification of individuals within externalizing (ADHD, conduct problems, oppositional defiant problems) and internalizing (anxiety problems, depressive problems, somatic problems) problems; (b) ROC curve for predicting the development of internalizing or externalizing problems (AUC = 0.79).

Since the models could accurately predict the development of each mental health problem separately, we next investigated whether CBCL data could also predict which mental health problem an individual would develop specifically. We randomly selected 255 individuals per group to balance the prediction data. We could not match all groups on demographic variables as we did for individual case–control classifications (for which we could select controls from a large sample), as the resulting sample was not sufficiently large. We then used multiclass classification with the same method as described above but without SHAP feature selection. Balanced accuracy was relatively low (24%) but higher than chance level (17%). Inspection of the confusion matrix (Figure 4a) indicated that the model mainly misclassified participants who are part of the cluster of internalizing problems (anxiety problems, depressive problems, and somatic problems) and between participants who are part of the cluster of externalizing problems (ADHD, conduct problems, and oppositional defiant problems) (Lahey et al., 2004).

To evaluate whether a model predicting the development of internalizing versus externalizing problems would perform better, we merged internalizing problems (anxiety problems, depressive problems, and somatic problems) and externalizing problems (ADHD, conduct problems, and oppositional defiant problems), and excluded participants with both an internalizing and externalizing problem. Classifying future internalizing and externalizing problems with 1106 individuals per group using a random forest classifier with SHAP feature selection yielded acceptable discrimination (AUC = 0.79) (Figure 4b). The top five features were ‘Disobedient at home’, ‘Argues a lot’, ‘Self-conscious’, ‘Easily jealous’, and ‘Breaks rules’, indicating that externalizing symptoms were most influential for distinguishing later internalizing from externalizing problems, as these features strongly aligned with observable behaviors commonly associated with externalizing problems. Separate classifications for internalizing problems (N = 2333) versus controls (N = 1642) yielded an AUC of 0.87 and for externalizing problems (N = 1220) versus controls (N = 781) an AUC of 0.93 (Supplementary Figures S8–S10). These results indicate that individuals from the internalizing, as well as externalizing problem groups can be better distinguished from individuals in the control group than individuals with later internalizing and externalizing problems can be distinguished from each other.

## Discussion

The results from this study show that the development of various mental health problems can be predicted in a large community sample of youth. Accurate models can be obtained with a standard clinical questionnaire (CBCL; AUCs between 0.71 and 0.87) and all features combined, but not with neuroimaging data only (AUCs < 0.6). Although it was not possible to differentiate between the development of specific mental health problems, the differentiation between internalizing and externalizing problems was acceptable (AUC = 0.79). The results were replicated in a second test set (AUCs >0.78), illustrating the robustness of the models. Together, these results demonstrate that subclinical symptoms are highly predictive for the development of later psychopathology and can differentiate between internalizing and externalizing problems. In contrast, neuroimaging features had little predictive value, indicating that variability in brain development is not informative at this age when brain development is at a turning point (Giedd et al., 1999).

We used SHAP feature selection to minimize the number of features to obtain good predictions. SHAP feature selection indicated that 10–29 items of the 119-item CBCL were selected for the predictive models. A simple summation of these items was sufficient to obtain good predictions, and the supplementary tables reflecting the sensitivity/specificity tradeoffs could be used to optimize the decision criterion. There was little overlap between items that are part of the original CBCL subscales and our predictive models. This indicates that there is no straightforward relationship between diagnostic symptoms (used in CBCL subscales) and prognostic markers (identified by our models). Instead, the predictive models rely on a broader set of behavioral features that extend beyond traditional diagnostic criteria.

Additionally, some predictive items were shared across symptom domains, reflecting the presence of overlapping behavioral markers and supporting the concept of comorbidity. This finding aligns with the broader discussion on comorbidity and is consistent with recent evidence suggesting shared underlying mechanisms across psychiatric disorders (Xie et al., 2023). These results emphasize the importance of considering both domain-specific and transdiagnostic markers when predicting symptom trajectories.

The CBCL was developed as a diagnostic scale, and there are only a few studies that used it to predict the onset of mental health problems. One study reported that the child behavior checklist-dysregulation profile (CBCL-DP) can predict high scores on DSM-5 personality traits 4 years later (De Caluwé, Decuyper, & De Clercq, 2013). And the internalizing broad-band scale of CBCL can predict subsequent agoraphobia, anxiety disorder, and social phobia, while the externalizing broad-band scale can predict major depression and disruptive behavior disorders in at-risk children (Petty et al., 2008). However, the number of cases was small, limiting the robustness of the findings, which may explain the unexpected predictivity of externalizing scales for depression. Besides the CBCL, other studies have used other questionnaires or electronic health care records to predict the development of mental health problems but with limited success (Rahman et al., 2020). Our predictive models show that SHAP-selected CBCL items are able to predict mental health problems well in 1–2 years, suggesting that parent reports of mental health symptoms are a useful screening tool.

Although the CBCL models could predict mental health status at 1–2 year follow-up, multi-label predictive models did not perform that well (accuracy = 24%) at distinguishing between different mental health problems. The confusion matrix indicated that the model mixed up different mental health problems that are known to cluster into internalizing and externalizing problems (Laceulle, Vollebergh, & Ormel, 2015). All internalizing problems had a high probability of being classified as somatic problems, while externalizing problems were more likely to be classified as ADHD and oppositional defiant problems. This could also be the result from our inability to exclude cases with disease comorbidity, as that would have reduced the sample size too much. This result reflects the comorbid nature of psychiatric disorders; depression, anxiety disorders, and somatic symptoms are correlated and often co-occur (Lallukka et al., 2019). Likewise, oppositional defiant disorder and conduct disorder are comorbid with ADHD, and oppositional defiant disorder is a predictor of conduct disorder onset (Burke, Rowe, & Boylan, 2014; Noordermeer et al., 2017). A model to distinguish between internalizing and externalizing problems showed acceptable discrimination. This implies that CBCL scores cannot only predict the development of specific mental health problems but that it can distinguish between the developments of internalizing or externalizing problems. However, the models performed better at distinguishing each clinical group from controls than at differentiating between internalizing and externalizing problems. This suggests that there is also symptom comorbidity between these problem domains. Symptom comorbidity may even be stronger than suggested by this result, as we excluded participants with both internalizing and externalizing problems beforehand. Thus while individuals who only develop internalizing or externalizing problems can be accurately distinguished, these problem domains also have inherent comorbidity within certain individuals.

When using other known risk factors reported previously in the literature, we found that sleep and family status were relatively important features for all predictive models. This is consistent with the results of previous studies using other clinical data, which identified that sleep quality, family history, and family environment were risk factors for the development of psychiatric disorders (Liu et al., 2017; Maniruzzaman, Shin, & Hasan, 2022). Although the model using CBCL outperformed the model using known risk factors, we chose to perform our follow-up analyses with this routinely used and well-validated instrument.

While CBCL items had high predictive value on their own, the neuroimaging measures only contributed to the prediction in combination with other features. This indicates that there may be an intricate interaction between the brain and behavior in predicting later problems. However, machine learning algorithms typically only select the most relevant features to optimize the prediction, which therefore does not provide information about their necessity. And vice versa, features that were not selected by the algorithm may just have been redundant and still relevant. The unimodal analysis showed CBCL items were sufficient while neuroimaging was not. One possibility for the limited contribution of neuroimaging is that the association between the brain and behavior is weak and its reliability poor (Liu, Abdellaoui, Verweij, & van Wingen, 2023). However, multivariate models can improve the predictive value of neuroimaging measures (Spisak, Bingel, & Wager, 2023), and previous studies have already demonstrated that neuroimaging can predict anxiety and depression in older children (Chavanne et al., 2023; Toenders et al., 2022). We therefore speculate that variability in brain structure and function in 9- to 10-year-old children may be more indicative of their current neurodevelopmental phase than related to their propensity to develop mental health problems.

Interestingly, the accuracy from our predictive models is higher than the accuracy of the CBCL for diagnosing DSM disorders as defined by clinical interviews (Krol et al., 2006). As the CBCL is used in clinical practice, this suggests that our CBCL-based predictive models may also be useful for patient care. Our instrument may help in identifying individuals at risk for developing clinically relevant mental health problems. These children may subsequently receive preventative interventions. These interventions are typically administered to entire schools or are targeted to children with the most symptoms. Targeted prevention is more successful, though the effect sizes are still small (Werner-Seidler et al., 2021). Personalizing prevention to individuals at risk may improve the preventative effect of these interventions.

Our findings also reveal notable differences in algorithm performance between models that include all features and models that include only CBCL items. For models including all features, the performance of different algorithms varied substantially. Logistic Regression consistently demonstrated robust results comparable to Random Forest, while SVM and MLP showed lower performance, particularly for domains such as Conduct Problems and Anxiety Problems. The lower performance may be attributed to the different data types included in these models. This may reflect challenges in effectively integrating and utilizing these diverse feature types.

In contrast, for models that included only CBCL items, all algorithms exhibited stable and competitive performance, with results comparable to Random Forest across all domains. This consistency highlights the robustness and predictive value of the CBCL features selected for these models. It suggests that the CBCL items we selected from SHAP are strongly associated with these mental health problems, enabling reliable predictions regardless of the algorithm used. While imaging and other data types may add complexity and variability to the models, CBCL-based models demonstrate that carefully selected features from a single modality can provide stable and interpretable predictions. This reinforces the utility of CBCL items as reliable predictors for mental health problems, even across different machine learning algorithms.

The current study is made possible by the large ABCD cohort study with repeated follow-up, which enabled the identification of sufficient children who were healthy at baseline and had clinically relevant mental health problems at follow-up. However, the use of this cohort comes with its limitations. First, it is not known whether the results from North American children can be generalized to children in other countries. Second, the children were between 9 and 10 years at baseline, and it is not clear whether similar predictions can be made for younger and older children. Third, the follow-up period was at the time of investigation restricted to 2 years. Children that did not yet develop clinically relevant problems may do so when they get older, which may also influence the predictive accuracy of our models. The ABCD cohort includes a 10-year follow-up, and models can be retrained for different ages and follow-up ranges once those data become available. Fourth, no self-reported symptom questionnaires across all psychiatric domains were available, as the CBCL only includes this for older children. Self-ratings may provide more insight into internalizing symptoms, and the results may therefore be inherently biased toward externalizing symptoms. Fifth, the limited performance of predictive models based on neuroimaging data could be related to the specific neuroimaging features that are present within the ABCD, as well as the arguably suboptimal modeling compared with behavioral data. Advanced metrics combined with more sophisticated algorithms such as deep learning could uncover more predictive information in future studies.

In conclusion, our results show that our learning-based models based on the CBCL can predict the development of various mental health problems in independent hold-out samples. The availability of accurate predictive models may enable screening and personalized prevention for at-risk children already before their symptoms require professional help. Furthermore, the identification of the most predictive features may provide clues for optimizing those prevention programs. Together, this will hopefully reduce the number of children who develop mental health problems.

## Supporting information

Hou et al. supplementary material Hou et al. supplementary material

## Data Availability

Data used in the preparation of this article can be obtained from the Adolescent Brain Cognitive Development (ABCD) Study (https://abcdstudy.org), held in the NIMH Data Archive (NDA).

## Supplementary material

The supplementary material for this articlecan be found at http://doi.org/10.1017/S003329172500087X.
