# Supplementary material for: Predicting the onset of mental health problems in adolescents
Source: Psychol Med. 2025 Apr 30;55:e128. doi: 10.1017/S003329172500087X (PMC12094648; doi:10.1017/S003329172500087X)
Supplement: Hou et al. supplementary material [file S003329172500087Xsup001.docx]

Supporting Information for

**Predicting the onset of mental disorders in adolescents**

Jiangyun Hou, Laurens van de Mortel, Arne Popma, Dirk Smit， Guido van Wingen

Corresponding author: Jiangyun Hou and Guido van Wingen

E-mail: [j.hou@amsterdamumc.nl](mailto:j.hou@amsterdamumc.nl)

E-mail: [g.a.vanwingen@amsterdamumc.nl](mailto:g.a.vanwingen@amsterdamumc.nl)

**This PDF file includes:**
Figs. S1 to S11

Tables. S1 to S20

**Fig. S1**. **The most** i**mportant features of the models using all data modalities to predict the onset of psychiatric disorders.**

(A) ADHD (B) Anxiety problems (C) Conduct problems (D) Depressive problems (E) Oppositional defiant problems (F) Somatic problems. (details of the top 10 features are shown in Supplementary Table S1-S6)


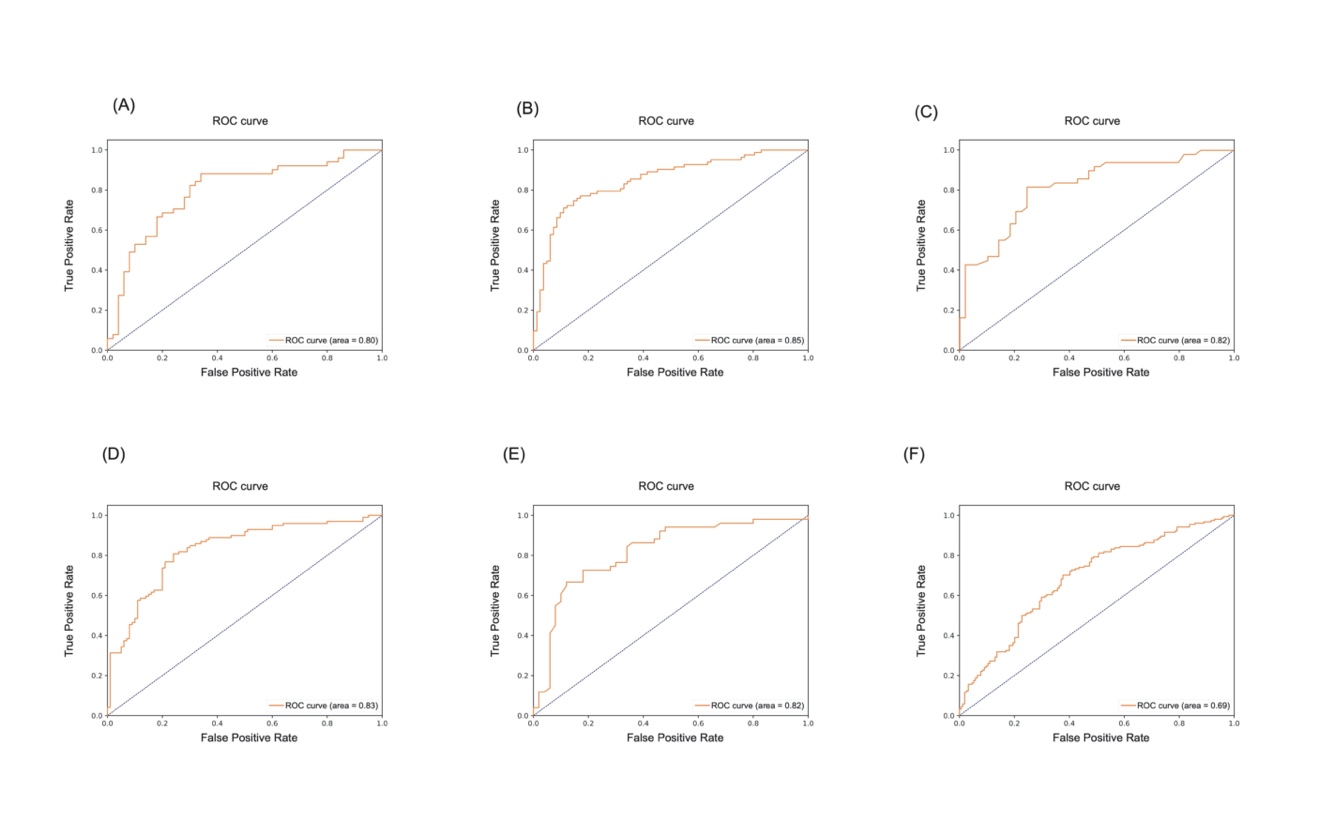


**Fig. S2. ROC curves for six models using multimodal data with important features from SHAP**

(A) ADHD: AUC=0.80 (B) Anxiety problems: AUC=0.85 (C) Conduct problems: AUC=0.82 (D) Depressive problems: AUC=0.83 (E) Oppositional defiant problems: AUC=0.82 (F) Somatic problems: AUC=0.69

_
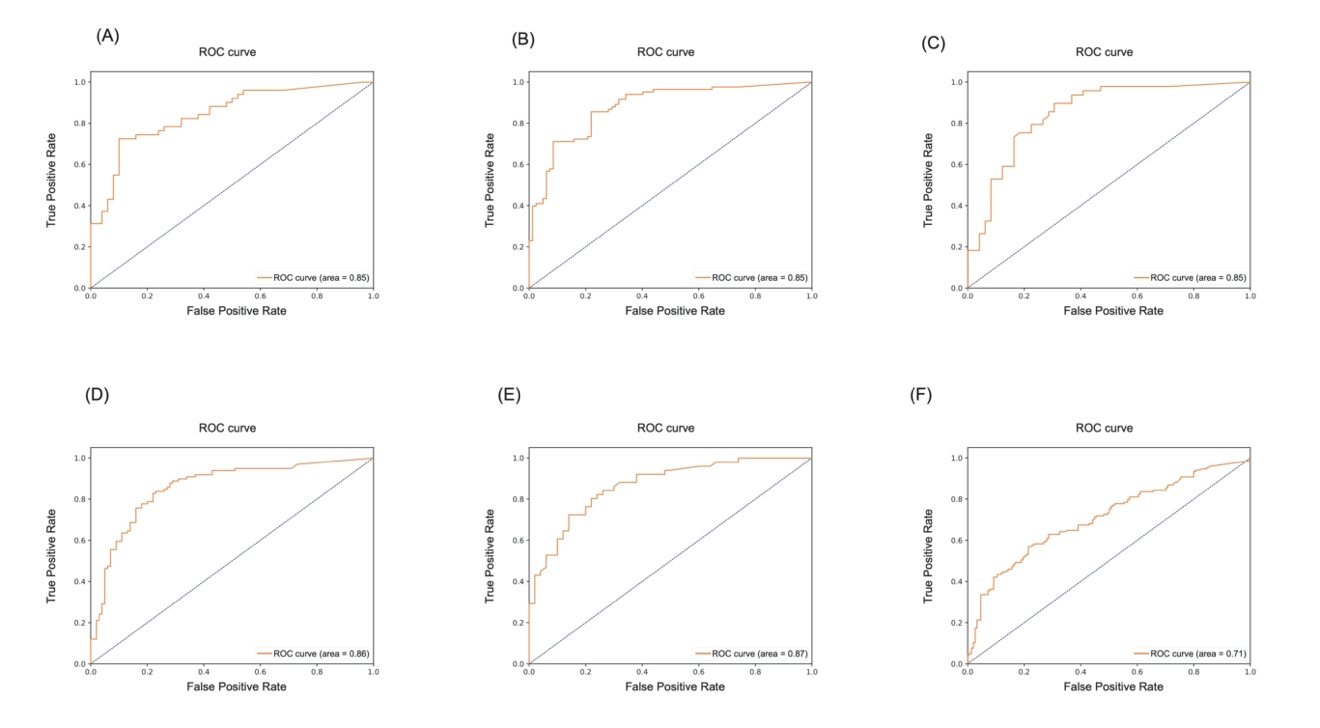
_

**Fig. S3. ROC curves for the prediction of psychiatric disorder development using the CBCL in the first test set.**

(A) ADHD: AUC=0.85 (B) Anxiety problems: AUC=0.85 (C) Conduct problems: AUC=0.85 (D) Depressive problems: AUC=0.86 (E) Oppositional defiant problems: AUC=0.87 (F) Somatic problems: AUC=0.71.


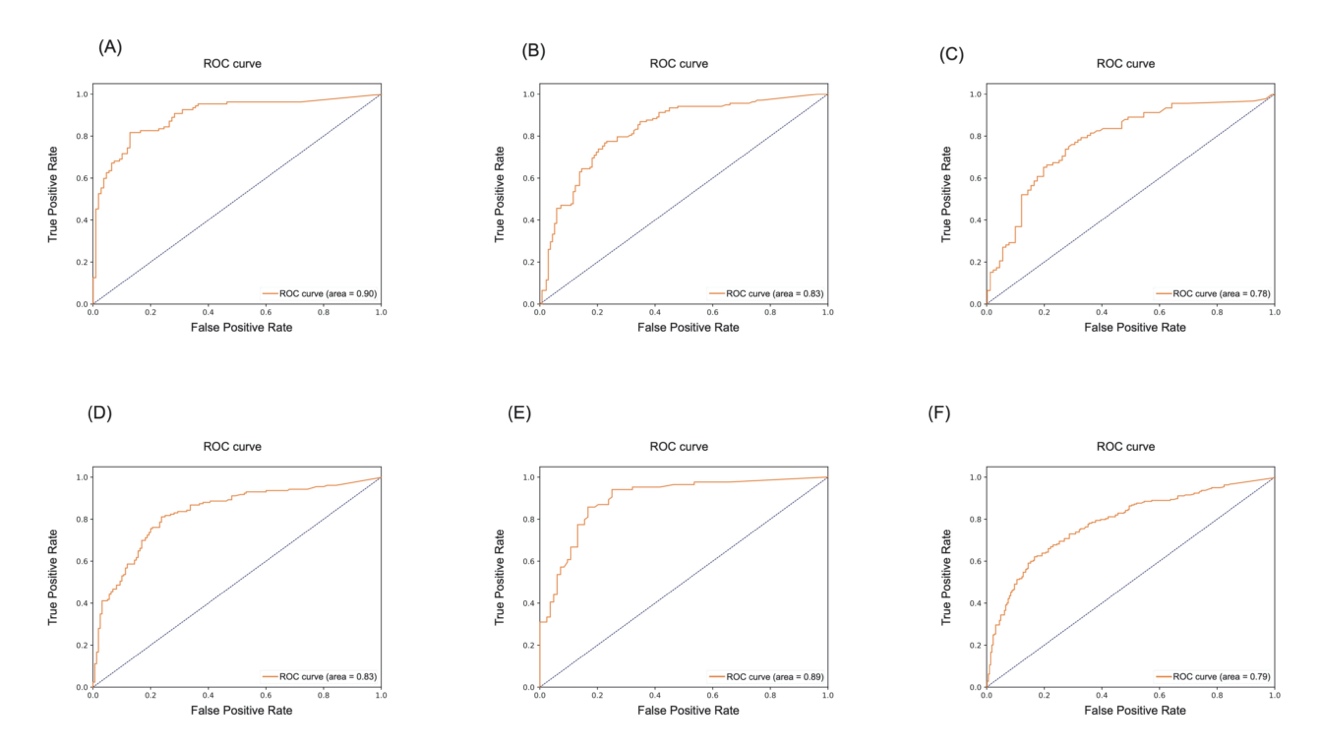


**Fig. S4. ROC curves for the prediction of psychiatric disorder development using the CBCL in the second test set**

(A) ADHD: AUC=0.90 (B) Anxiety problems: AUC=0.83 (C) Conduct problems: AUC=0.78 (D) Depressive problems: AUC=0.83 (E) Oppositional defiant problems: AUC=0.89 (F) Somatic problems: AUC=0.79.

**Fig. S5.** **ROC curve of CBCL total score of the first test set**

(A) ADHD: AUC=0.931 (B) Anxiety problems: AUC=0.909 (C) Conduct problems: AUC=0.926 (D) Depressive problems: AUC=0.910 (E) Oppositional defiant problems: AUC=0.947 (F) Somatic problems: AUC=0.779.

**Fig. S6.** **ROC curve of CBCL total score** **of the second test set**

(A) ADHD: AUC=0.944 (B) Anxiety problems: AUC=0.881 (C) Conduct problems: AUC=0.849 (D) Depressive problems: AUC=0.881 (E) Oppositional defiant problems: AUC=0.944 (F) Somatic problems: AUC=0.813.


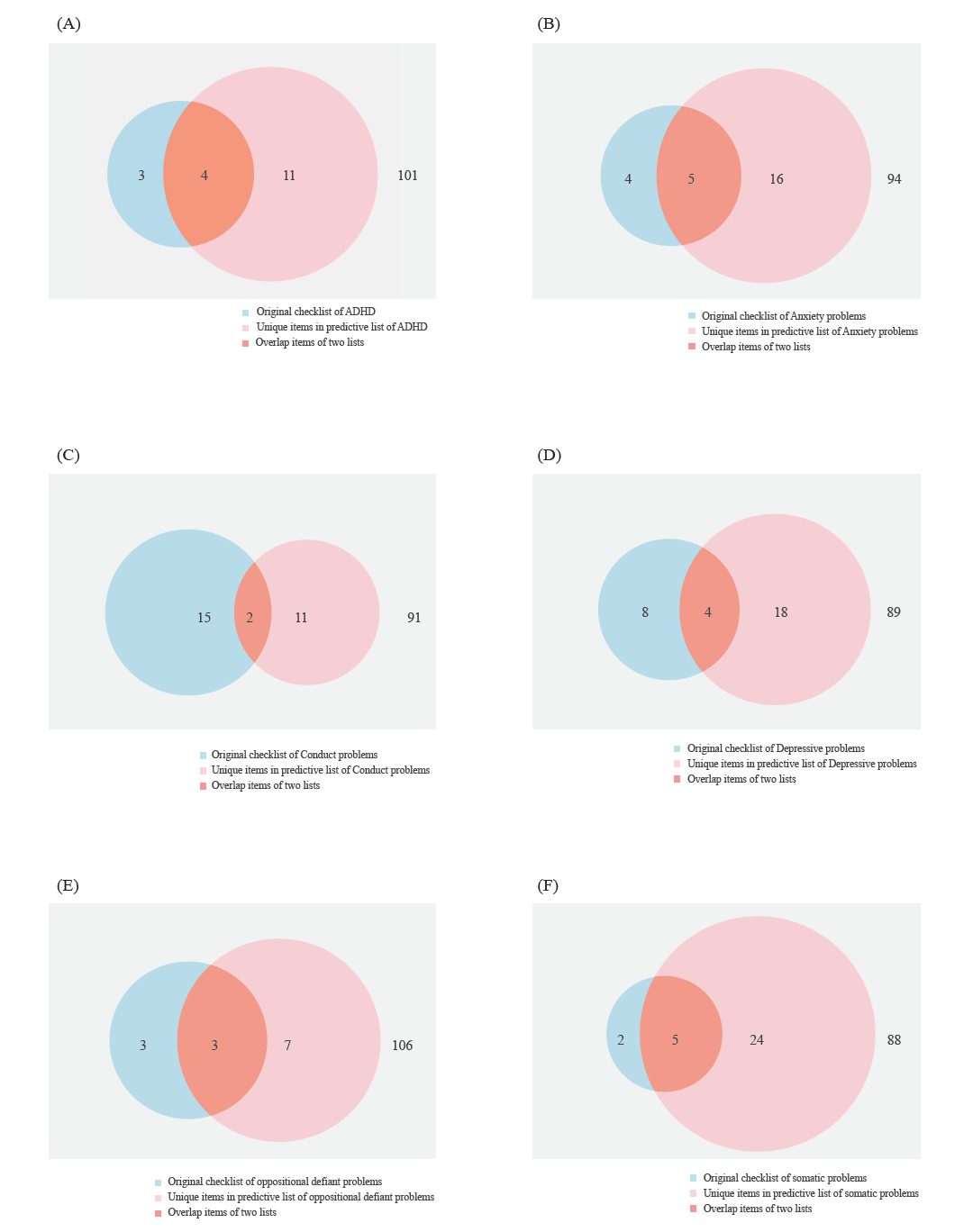


**Fig. S7. The Venn diagram from original checklist and predictive list.**

(A) the Venn diagram of ADHD (Jaccard index= 0.22) (B) the Venn diagram of Anxiety problems (Jaccard index= 0.20) (C) the Venn diagram of Conduct problems (Jaccard index= 0.07) (D) the Venn diagram of Depressive problems (Jaccard index= 0.13) (E) the Venn diagram of Oppositional defiant problems (Jaccard index= 0.23) (F) the Venn diagram of Somatic problems (Jaccard index= 0.16) (blue: the CBCL items for diagnosis; red: the CBCL items for prognosis).


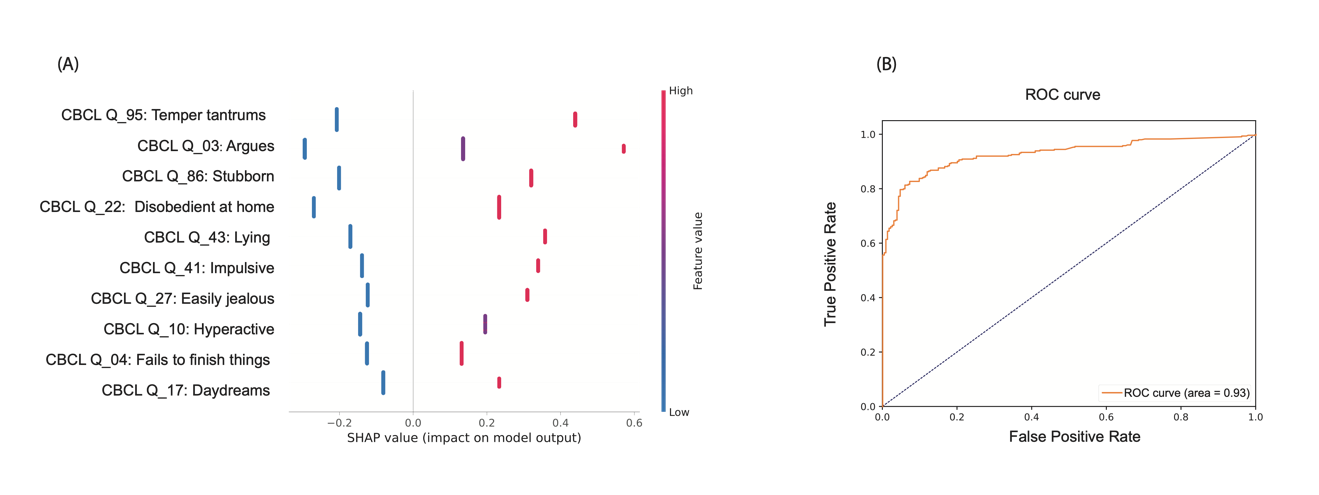


**Fig. S8. Results of externalizing vs controls model**

(A) the top 10 important features of externalizing vs controls model from SHAP; (B) ROC curve of externalizing model.

**
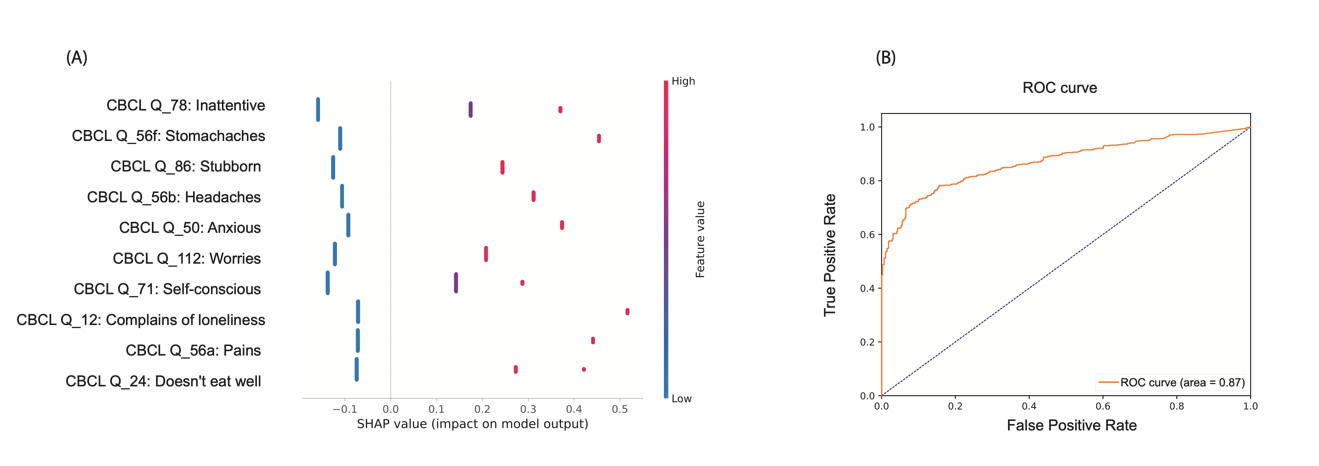
**

**Fig. S9. Results of internalizing vs controls model**

(A) the top 10 important features of internalizing vs controls model from SHAP; (B) ROC curve of internalizing model.

**Fig. S10. The top 10 important features of internalizing vs externalizing model from SHAP.**

**
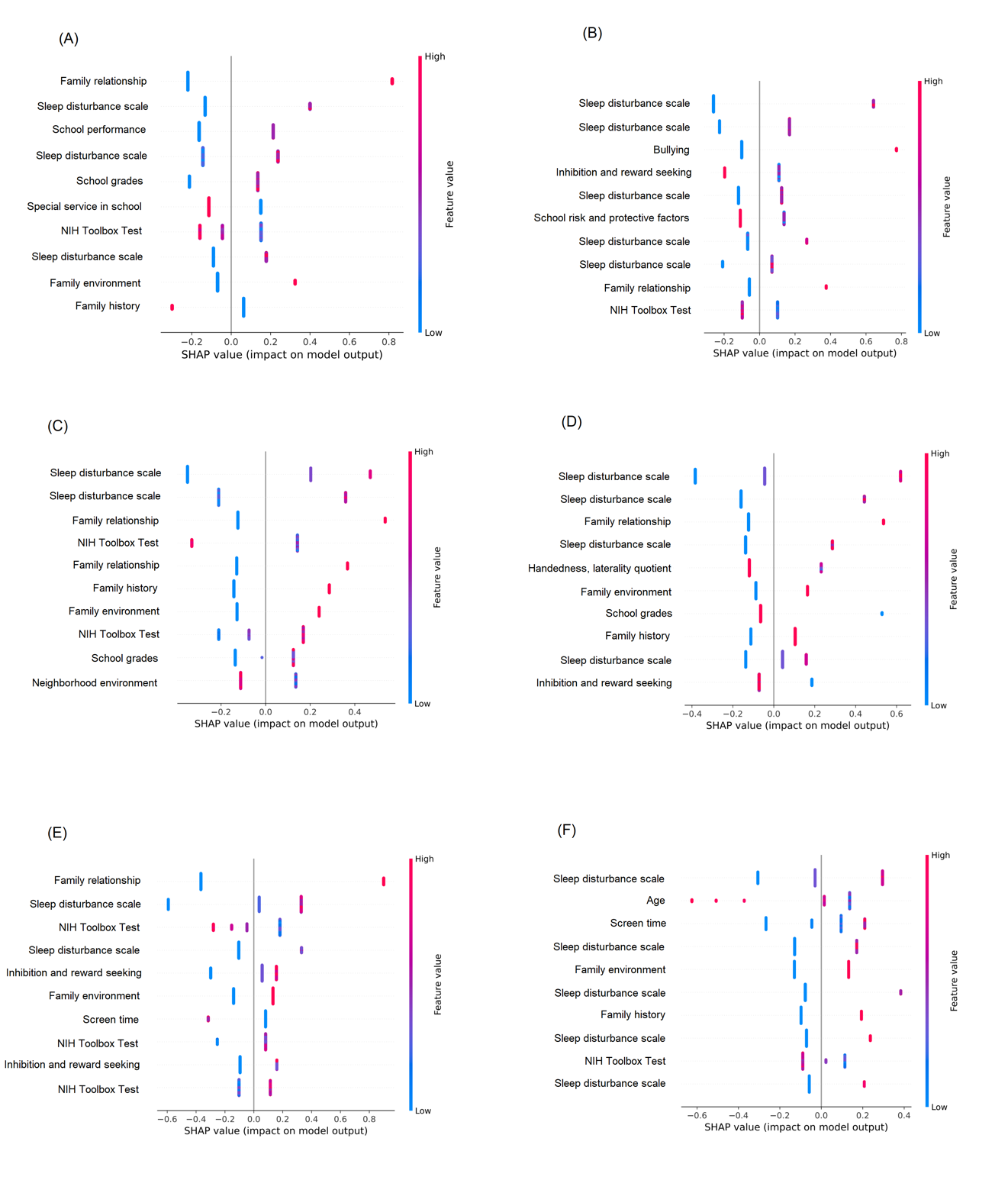
**

**Fig. S11. The most** i**mportant features of the models using risk factors data to predict the onset of psychiatric disorders.**

(A) ADHD (B) Anxiety problems (C) Conduct problems (D) Depressive problems (E) Oppositional defiant problems (F) Somatic problems.

**Table S1. Top 10 important features of ADHD with all features.**

|  | Important features | Short name | Data source |
| --- | --- | --- | --- |
| 1. | can't sit still, restless, or hyperactive | cbcl_q10_p | CBCL raw score |
| 2. | Inattentive or easily distracted | cbcl_q78_p | CBCL raw score |
| 3. | Argues a lot | cbcl_q03_p | CBCL raw score |
| 4. | Mean beta weight for MID reward positive versus negative feedback contrast in left hemisphere cortical Destrieux ROI inferior segment of the circular sulcus of the insula | midabwdp344 | beta weights for MID task fMRI in cortical (Destrieux) ROIs averaged across runs |
| 5. | Can't concentrate, can't pay attention for long | cbcl_q08_p | CBCL raw score |
| 6. | Mean beta weight for nBack run 2 emotion versus neutral face contrast in ASEG ROI left-cerebellum-cortex | tfmri_nback_r2_185 | beta weights for run 2 nBack task fMRI in subcortical (aseg) ROIs |
| 7. | Mean beta weight for run 2 nBack 2 back condition in left hemisphere cortical Destrieux ROI superior frontal sulcus | tfnbr2bwdp_201 | beta weights for run 2 nBack task fMRI in cortical (Desikan) ROIs |
| 8. | Demands a lot of attention | cbcl_q19_p | CBCL raw score |
| 9. | Temporal variance in left hemisphere cortical Destrieux ROI triangular part of the inferior frontal gyrus | mrirsfd14 | rsfMRI Destrieux |
| 10. | Mean beta weight for run 2 SST correct stop versus correct go contrast in left hemisphere cortical Destrieux ROI inferior temporal sulcus | tfsstr2bwdp_220 | beta weights for run 2 SST task fMRI in cortical (Destrieux) ROIs |

**Table S2. Top 10 important features of anxiety problems with all features.**

|  | Important features | Short name | Data source |
| --- | --- | --- | --- |
| 1. | Worries | cbcl_q112_p | CBCL raw score |
| 2. | Self-conscious or easily embarrassed | cbcl_q71_p | CBCL raw score |
| 3. | Too fearful or anxious | cbcl_q50_p | CBCL raw score |
| 4. | Beta weight for MID run1 anticipation of large versus small loss contrast in cortical Destrieux ROI left hemisphere transverse frontopolar gyri and sulci | tfmrimr1_1186 | beta weights for run1 MID task fMRI in cortical (Destrieux) ROIs |
| 5. | (CortContrast-White - CortContrast-Gray) / ((CortContrast-White plus CortContrast-Gray)/2) of T1 weighted image for APARC ROI rh-parsorbitalis | smri_t1wcnt_cdk_parsobisrh | sMRI in cortical (aparc) ROIs |
| 6. | Inattentive or easily distracted | cbcl_q78_p | CBCL raw score |
| 7. | Beta weight for MID run2 reward positive versus negative feedback contrast in ASEG ROI left-thalamus-proper | tfmri_mr2_rpvnfb_bscs_tplh | beta weights for run2 MID task fMRI in subcortical (aseg) ROIs |
| 8. | Mean beta weight for nBack positive face versus neutral face contrast in ASEG ROI left-lateral-ventricle | tfmri_nback_all_242 | beta weights for nback task fMRI in subcortical (aseg) ROIs averaged across runs |
| 9. | Easily jealous | cbcl_q27_p | CBCL raw score |
| 10. | The child feels anxious or afraid when falling asleep. | sleepdisturb5_p | sleep disturbance scale |

**Table S3. Top 10 important features of conduct problems with all features.**

|  | Important features | Short name | Data source |
| --- | --- | --- | --- |
| 1. | Breaks rules at home, school or elsewhere | cbcl_q28_p | CBCL raw score |
| 2. | Disobedient at home | cbcl_q22_p | CBCL raw score |
| 3. | Mean beta weight for nBack 2 back condition in left hemisphere cortical Destrieux ROI supramarginal gyrus | tfabwdp_174 | Beta weights for nBack task fMRI in cortical (Destrieux) ROIs |
| 4. | The child goes to bed reluctantly. | sleepdisturb3_p | sleep disturbance scale |
| 5. | Argues a lot | cbcl_q03_p | CBCL raw score |
| 6. | Fails to finish things they start | cbcl_q04_p | CBCL raw score |
| 7. | restricted normalized total diffusion gray-white contrast Destrieux cortical parcellation left hemisphere lateral occipito-temporal sulcus | dmri_rsirntgwc_cdx_sotllh | Diffusion Tensor Imaging measures in cortical (Destrieux) ROIs |
| 8. | Mean beta weight for run 1 SST correct stop versus correct go contrast in left hemisphere cortical Destrieux ROI subcallosal gyrus | tfsstr1bwdp_180 | Beta weights for run1 SST task fMRI in cortical (Destrieux) ROIs |
| 9. | Standard error of the mean for run 2 nBack emotional face versus neutral face contrast in left hemisphere cortical Destrieux ROI medial occipito-temporal sulcus and lingual sulcus | tfnbr2dp_949 | Beta weights for run2 nBack task fMRI in cortical (Destrieux) ROIs |
| 10. | Cortical volume in mm^3 for right hemisphere cortical Destrieux ROI calcarine sulcu | mrisdp_571 | sMRI |

**Table S4. Top 10 important features of depressive problems with all features.**

|  | Important features | Short name | Data source |
| --- | --- | --- | --- |
| 1. | The child has difficulty getting to sleep at night. | sleepdisturb4_p | sleep disturbance scale |
| 2. | Fails to finish things they start | cbcl_q04_p | CBCL raw score |
| 3. | Beta weight for MID run1 anticipation of large versus small loss contrast in APARC ROI lh-entorhinal | tfmri_mr1_alvsl_bcdk_ehinallh | Beta weights for run1 MID task fMRI in cortical (aparc) ROIs |
| 4. | restricted normalized isotropic diffusion peri-cortical white matter Desikan cortical parcellation right hemisphere caudalanteriorcingulate | dmri_rsirniwm_cdk_cacrh | Diffusion Tensor Imaging measures in cortical (Destrieux) ROIs |
| 5. | Would rather be alone than with others | cbcl_q42_p | CBCL raw score |
| 6. | Stubborn, sullen, or irritable | cbcl_q86_p | CBCL raw score |
| 7. | Sulks a lot | cbcl_q88_p | sleep disturbance scale |
| 8. | Argues a lot | cbcl_q03_p | CBCL raw score |
| 9. | Mean beta weight for nBack place condition in right hemisphere cortical Destrieux ROI posterior-dorsal part of the cingulate gyrus | tfabwdp_379 | Beta weights for nBack task fMRI in cortical (Destrieux) ROIs |
| 10. | free normalized isotropic diffusion gray-white contrast Destrieux cortical parcellation right hemisphere transverse temporal sulcus | dmri_rsifnigwc_cdx_sttrh | Diffusion Tensor Imaging measures in cortical (Destrieux) ROIs |

**Table S5. Top 10 important features of oppositional defiant problems with all features.**

|  | Important features | Short name | Data source |
| --- | --- | --- | --- |
| 1. | Temper tantrums or hot temper | cbcl_q95_p | CBCL raw score |
| 2. | Argues a lot | cbcl_q03_p | CBCL raw score |
| 3. | Disobedient at home | cbcl_q22_p | CBCL raw score |
| 4. | Can't sit still, restless, or hyperactive | cbcl_q10_p | CBCL raw score |
| 5. | Mean beta weight for run 1 MID anticipation of large reward versus neutral contrast in right hemisphere cortical Destrieux ROI superior part of the precentral sulcus | tfmrimr1_735 | Beta weights for run 1 MID task fMRI in cortical (Destrieux) ROIs |
| 6. | Mean beta weight for run 1 SST incorrect go versus incorrect stop contrast in right hemisphere cortical Destrieux ROI orbital gyri | tfsstr1bwdp_986 | Beta weights for run 1 SST task fMRI in cortical (Destrieux) ROIs |
| 7. | Mean beta weight for nBack emotion condition in left hemisphere cortical Destrieux ROI long insular gyrus and central sulcus of the insula | tfabwdp_461 | Beta weights for SST task fMRI in cortical (Destrieux) ROIs averaged across runs |
| 8. | Mean beta weight for nBack emotion condition in left hemisphere cortical Destrieux ROI inferior occipital gyrus and sulcus | tfabwdp_446 | Beta weights for SST task fMRI in cortical (Destrieux) ROIs averaged across runs |
| 9. | Mean beta weight for run 2 SST incorrect stop versus correct go contrast in left hemisphere cortical Destrieux ROI horizontal ramus of the anterior segment of the lateral sulcus | tfsstr2bwdp_335 | Beta weights for run 2 SST task fMRI in subcortical (aseg) and cortical (Desikan aparc) ROIs (part 2) |
| 10. | Breaks rules at home, school or elsewhere | cbcl_q28_p | CBCL raw score |

**Table S6. Top 10 important features of somatic problems with all features.**

|  | Important features | Short name | Data source |
| --- | --- | --- | --- |
| 1. | Headaches | cbcl_q56b_p | CBCL raw score |
| 2. | The child goes to bed reluctantly. | sleepdisturb3_p | sleep disturbance scale |
| 3. | Self-conscious or easily embarrassed | cbcl_q71_p | CBCL raw score |
| 4. | Argues a lot | cbcl_q03_p | CBCL raw score |
| 5. | the child has difficulty getting to sleep at night | Age in months at the time of the interview/test/sampling/imaging. | Age |
| 6. | Whining | cbcl_q109_p | CBCL raw score |
| 7. | You have observed the child talking in their sleep. | sleepdisturb18_p | sleep disturbance scale |
| 8. | Mean beta weight for nBack 0 back condition in APARC ROI lh-postcentral | tfmri_nback_all_291 | beta weights for nBack task fMRI in cortical (Destrieux) ROIs averaged across runs |
| 9. | Mean beta weight for nBack positive face versus neutral face contrast in ASEG ROI right-amygdala | tfmri_nback_all_268 | beta weights for nBack task fMRI in subcortical (aseg) ROIs averaged across runs |
| 10. | Beta weight for MID run1 anticipation of large reward versus small reward contrast in cortical Destrieux ROI right hemisphere subcallosal gyrus | tfmrimr1_994 | beta weights for run1 MID task fMRI in cortical (Destrieux) ROIs |

**Table S7. Important items in models with CBCL items only (green background: same items as original CBCL DSM-5 checklist).**

|  | ADHD | Anxiety  problems | Conduct  problems | Depressive  problems | Oppositional defiant problems | Somatic  problems |
| --- | --- | --- | --- | --- | --- | --- |
| Acts too young for their age |  | √ |  |  |  |  |
| Drinks alcohol without parents' approval |  |  |  |  |  |  |
| Argues a lot | √ |  | √ | √ | √ | √ |
| Fails to finish things they start |  |  | √ | √ |  |  |
| There is very little they enjoy |  |  |  |  |  |  |
| Bowel movements outside toilet |  |  |  |  |  |  |
| Bragging, boasting |  |  |  |  |  |  |
| Can't concentrate, can't pay attention for long | √ |  |  |  |  | √ |
| Can't get their mind off certain thoughts; obsessions |  |  | √ |  | √ |  |
| Can't sit still, restless, or hyperactive N | √ |  |  |  | √ | √ |
| Clings to adults or too dependent |  |  |  |  |  |  |
| Complains of loneliness |  |  |  |  |  | √ |
| Confused or seems to be in a fog |  |  |  |  |  |  |
| Cries a lot |  |  |  | √ |  |  |
| Cruel to animals |  |  |  |  |  |  |
| Cruelty, bullying, or meanness to others |  |  |  |  |  |  |
| Daydreams or gets lost in their thoughts | √ | √ | √ | √ |  |  |
| Deliberately harms self or attempts suicide |  |  |  |  |  |  |
| Demands a lot of attention | √ |  |  | √ | √ | √ |
| Destroys their own things |  |  |  |  |  |  |
| Destroys things belonging to their family or others |  |  |  |  |  |  |
| Disobedient at home |  |  | √ |  | √ | √ |
| Disobedient at school |  |  |  |  |  |  |
| Doesn't eat well |  | √ |  | √ |  |  |
| Doesn't get along with other kids |  |  |  |  |  |  |
| Doesn't seem to feel guilty after misbehaving |  |  |  |  |  | √ |
| Easily jealous | √ | √ | √ |  | √ | √ |
| Breaks rules at home, school or elsewhere |  |  | √ | √ | √ |  |
| Fears certain animals, situations, or places, other than school |  | √ |  |  |  |  |
| Fears going to school |  |  |  |  |  |  |
| Fears they might think or do something bad |  |  |  |  |  | √ |
| Feels they have to be perfect |  | √ |  | √ |  |  |
| Feels or complains that no one loves them |  |  |  |  |  |  |
| Feels others are out to get them |  |  |  |  |  |  |
| Feels worthless or inferior |  | √ |  |  |  |  |
| Gets hurt a lot, accident prone |  |  |  |  |  |  |
| Gets in many fights |  |  |  |  |  |  |
| Gets teased a lot |  |  |  |  |  |  |
| Hangs around with others who get in trouble |  |  |  |  |  |  |
| Hears sound or voices that aren't there |  |  |  |  |  |  |
| Impulsive or acts without thinking | √ |  |  |  |  |  |
| Would rather be alone than with others |  |  | √ | √ |  |  |
| Lying or cheating | √ |  | √ | √ | √ |  |
| Bites fingernails |  |  |  |  |  |  |
| Nervous, highstrung, or tense |  | √ |  | √ |  |  |
| Nervous movements or twitching |  |  |  |  |  |  |
| Nightmares |  |  |  | √ |  | √ |
| Not liked by other kids |  |  |  |  |  |  |
| Constipated, doesn't move bowels |  |  |  |  |  | √ |
| Too fearful or anxious |  | √ |  |  |  | √ |
| Feels dizzy or lightheaded |  |  |  |  |  |  |
| Feels too guilty |  |  |  |  |  |  |
| Overeating Come |  |  |  | √ |  | √ |
| Overtired without good reason |  |  |  |  |  |  |
| Overweight |  |  |  |  |  |  |
| Aches or pains (not stomach or headaches) |  | √ |  | √ |  | √ |
| Headaches | √ |  | √ |  |  | √ |
| Nausea, feels sick |  |  |  |  |  | √ |
| Problems with eyes (not if corrected by glasses) |  |  |  |  |  |  |
| Rashes or other skin problems |  |  |  |  |  | √ |
| Stomachaches Dolores |  | √ |  | √ |  | √ |
| Vomiting, throwing up |  |  |  |  |  |  |
| Other (physical problems without known physical cause) |  |  |  |  |  |  |
| Physically attacks people |  |  |  |  |  |  |
| Picks nose, skin, or other parts of body |  |  |  |  |  | √ |
| Plays with own sex parts in public |  |  |  |  |  |  |
| Plays with own sex parts too much |  |  |  |  |  |  |
| Poor school work | √ |  |  |  |  |  |
| Poorly coordinated or clumsy |  |  |  |  |  | √ |
| Prefers being with older kids |  |  |  |  |  |  |
| Prefers being with younger kids |  | √ |  |  |  | √ |
| Refuses to talk |  |  |  |  |  |  |
| Repeats certain acts over and over; compulsions |  |  |  |  |  |  |
| Runs away from home |  |  |  |  |  |  |
| Screams a lot |  | √ |  |  |  |  |
| Secretive, keeps things to self |  |  |  | √ |  |  |
| Sees things that aren't there |  |  |  |  |  |  |
| Self-conscious or easily embarrassed |  | √ |  |  |  | √ |
| Sets fires |  |  |  |  |  |  |
| Sexual problems |  |  |  |  |  |  |
| Showing off or clowning |  |  |  |  |  |  |
| Too shy or timid |  | √ |  |  |  |  |
| Sleeps less than most kids |  |  |  | √ |  |  |
| Sleeps more than most kids during day and/or night |  |  |  |  |  |  |
| Inattentive or easily distracted | √ | √ |  |  | √ |  |
| Speech problem |  |  |  |  |  | √ |
| Stares blankly |  |  |  |  |  | √ |
| Steals at home |  |  |  |  |  |  |
| Steals outside the home |  |  |  |  |  |  |
| Stores up too many things they don't need |  | √ |  |  |  | √ |
| Strange behavior |  |  |  |  |  |  |
| Strange ideas |  |  |  |  |  |  |
| Stubborn, sullen, or irritable | √ |  |  | √ |  |  |
| Sudden changes in mood or feelings | √ |  |  |  |  | √ |
| Sulks a lot |  |  |  | √ |  |  |
| Suspicious |  |  |  |  |  |  |
| Swearing or obscene language |  |  |  |  |  |  |
| Talks about killing self |  |  |  |  |  |  |
| Talks or walks in sleep |  |  |  |  |  | √ |
| Talks too much |  | √ |  |  |  |  |
| Teases a lot |  |  | √ |  |  |  |
| Temper tantrums or hot temper |  | √ | √ | √ | √ | √ |
| Thinks about sex too much |  |  |  |  |  |  |
| Threatens people |  |  |  |  |  |  |
| Thumb-sucking |  |  |  |  |  |  |
| Smokes, chews, or sniffs tobacco |  |  |  |  |  |  |
| Trouble sleeping |  |  |  | √ |  |  |
| Truancy, skips school |  |  |  |  |  |  |
| Underactive, slow moving, or lacks energy |  |  |  |  |  |  |
| Unhappy, sad, or depressed |  |  |  |  |  |  |
| Unusually loud |  |  |  |  |  |  |
| Uses drugs for non medical purposes (don't include alcohol or tobacco) |  |  |  |  |  |  |
| Vandalism |  |  |  |  |  |  |
| Wets self during the day |  |  |  |  |  |  |
| Wets the bed |  |  |  |  |  |  |
| Whining | √ | √ |  |  |  | √ |
| Wishes to be of opposite sex |  |  |  |  |  |  |
| Withdrawn, doesn't get involved with others |  |  |  |  |  |  |
| Worries | √ | √ | √ | √ |  |  |

**Table S8. Coordinates of the Curve of sum scores of important features of ADHD in the first test set.**

| **Coordinates of the Curve** | | |
| --- | --- | --- |
| Test Result Variable(s): sum | | |
| Positive if Greater Than or Equal To^a^ | Sensitivity | Specificity |
| -1.0000 | 1 | 0 |
| .5000 | 0.982 | 0.22 |
| 1.5000 | 0.976 | 0.387 |
| 2.5000 | 0.929 | 0.56 |
| 3.5000 | 0.893 | 0.708 |
| 4.5000 | 0.833 | 0.827 |
| 5.5000 | 0.78 | 0.875 |
| 6.5000 | 0.708 | 0.917 |
| 7.5000 | 0.607 | 0.952 |
| 8.5000 | 0.488 | 0.958 |
| 9.5000 | 0.417 | 0.988 |
| 10.5000 | 0.345 | 0.994 |
| 11.5000 | 0.274 | 1 |
| 12.5000 | 0.149 | 1 |
| 13.5000 | 0.095 | 1 |
| 14.5000 | 0.065 | 1 |
| 15.5000 | 0.024 | 1 |
| 16.5000 | 0.012 | 1 |
| 17.5000 | 0.006 | 1 |
| 19.0000 | 0 | 1 |

**Table S9. Coordinates of the Curve of sum scores of important features of anxiety problems in the first test set.**

| **Coordinates of the Curve** | | |
| --- | --- | --- |
| Test Result Variable(s): sum | | |
| Positive if Greater Than or Equal To^a^ | Sensitivity | Specificity |
| -1.0000 | 1 | 0 |
| .5000 | 0.975 | 0.247 |
| 1.5000 | 0.96 | 0.393 |
| 2.5000 | 0.927 | 0.553 |
| 3.5000 | 0.902 | 0.684 |
| 4.5000 | 0.858 | 0.753 |
| 5.5000 | 0.76 | 0.829 |
| 6.5000 | 0.698 | 0.887 |
| 7.5000 | 0.622 | 0.913 |
| 8.5000 | 0.542 | 0.96 |
| 9.5000 | 0.447 | 0.978 |
| 10.5000 | 0.324 | 0.989 |
| 11.5000 | 0.262 | 0.989 |
| 12.5000 | 0.185 | 0.996 |
| 13.5000 | 0.127 | 1 |
| 14.5000 | 0.087 | 1 |
| 15.5000 | 0.047 | 1 |
| 16.5000 | 0.033 | 1 |
| 17.5000 | 0.018 | 1 |
| 19.0000 | 0.011 | 1 |
| 20.5000 | 0.007 | 1 |
| 21.5000 | 0.004 | 1 |
| 23.0000 | 0 | 1 |

**Table S10. Coordinates of the Curve of sum scores of important features of conduct problems in the first test set.**

| **Coordinates of the Curve** | | |
| --- | --- | --- |
| Test Result Variable(s): sum | | |
| Positive if Greater Than or Equal To^a^ | Sensitivity | Specificity |
| -1.0000 | 1 | 0 |
| .5000 | 0.982 | 0.313 |
| 1.5000 | 0.963 | 0.485 |
| 2.5000 | 0.92 | 0.638 |
| 3.5000 | 0.865 | 0.73 |
| 4.5000 | 0.779 | 0.828 |
| 5.5000 | 0.706 | 0.896 |
| 6.5000 | 0.583 | 0.957 |
| 7.5000 | 0.454 | 0.982 |
| 8.5000 | 0.27 | 0.988 |
| 9.5000 | 0.196 | 1 |
| 10.5000 | 0.135 | 1 |
| 11.5000 | 0.086 | 1 |
| 12.5000 | 0.055 | 1 |
| 13.5000 | 0.037 | 1 |
| 14.5000 | 0.012 | 1 |
| 15.5000 | 0.006 | 1 |
| 17.0000 | 0 | 1 |

**Table S11. Coordinates of the Curve of sum scores of important features of depressive problems in the first test set.**

| **Coordinates of the Curve** | | |
| --- | --- | --- |
| Test Result Variable(s): sum | | |
| Positive if Greater Than or Equal To^a^ | Sensitivity | Specificity |
| -1.0000 | 1 | 0 |
| .5000 | 0.982 | 0.202 |
| 1.5000 | 0.952 | 0.366 |
| 2.5000 | 0.931 | 0.559 |
| 3.5000 | 0.891 | 0.713 |
| 4.5000 | 0.846 | 0.819 |
| 5.5000 | 0.761 | 0.858 |
| 6.5000 | 0.662 | 0.924 |
| 7.5000 | 0.586 | 0.943 |
| 8.5000 | 0.471 | 0.955 |
| 9.5000 | 0.393 | 0.97 |
| 10.5000 | 0.305 | 0.982 |
| 11.5000 | 0.23 | 0.988 |
| 12.5000 | 0.16 | 0.994 |
| 13.5000 | 0.082 | 1 |
| 14.5000 | 0.054 | 1 |
| 15.5000 | 0.03 | 1 |
| 17.0000 | 0.009 | 1 |
| 18.5000 | 0.003 | 1 |
| 20.0000 | 0 | 1 |

**Table S12. Coordinates of the Curve of sum scores of important features of oppositional defiant problems in the first test set.**

| **Coordinates of the Curve** | | |
| --- | --- | --- |
| Test Result Variable(s): sum | | |
| Positive if Greater Than or Equal To^a^ | Sensitivity | Specificity |
| -1.0000 | 1 | 0 |
| .5000 | 0.994 | 0.357 |
| 1.5000 | 0.952 | 0.589 |
| 2.5000 | 0.94 | 0.75 |
| 3.5000 | 0.863 | 0.833 |
| 4.5000 | 0.756 | 0.911 |
| 5.5000 | 0.637 | 0.94 |
| 6.5000 | 0.482 | 0.964 |
| 7.5000 | 0.387 | 0.976 |
| 8.5000 | 0.28 | 0.982 |
| 9.5000 | 0.214 | 0.994 |
| 10.5000 | 0.113 | 1 |
| 11.5000 | 0.065 | 1 |
| 12.5000 | 0.036 | 1 |
| 13.5000 | 0.03 | 1 |
| 14.5000 | 0.018 | 1 |
| 15.5000 | 0.012 | 1 |
| 17.0000 | 0 | 1 |

**Table S13. Coordinates of the Curve of sum scores of important features of somatic problems in the first test set.**

| **Coordinates of the Curve** | | |
| --- | --- | --- |
| Test Result Variable(s): sum | | |
| Positive if Greater Than or Equal To^a^ | Sensitivity | Specificity |
| -1.0000 | 1 | 0 |
| .5000 | 0.977 | 0.168 |
| 1.5000 | 0.914 | 0.281 |
| 2.5000 | 0.864 | 0.437 |
| 3.5000 | 0.776 | 0.563 |
| 4.5000 | 0.68 | 0.671 |
| 5.5000 | 0.612 | 0.768 |
| 6.5000 | 0.515 | 0.828 |
| 7.5000 | 0.439 | 0.867 |
| 8.5000 | 0.374 | 0.91 |
| 9.5000 | 0.308 | 0.93 |
| 10.5000 | 0.273 | 0.959 |
| 11.5000 | 0.226 | 0.975 |
| 12.5000 | 0.199 | 0.99 |
| 13.5000 | 0.156 | 0.996 |
| 14.5000 | 0.113 | 0.998 |
| 15.5000 | 0.088 | 1 |
| 16.5000 | 0.074 | 1 |
| 17.5000 | 0.06 | 1 |
| 18.5000 | 0.049 | 1 |
| 19.5000 | 0.033 | 1 |
| 20.5000 | 0.025 | 1 |
| 21.5000 | 0.021 | 1 |
| 22.5000 | 0.018 | 1 |
| 23.5000 | 0.012 | 1 |
| 24.5000 | 0.008 | 1 |
| 26.5000 | 0.006 | 1 |
| 29.5000 | 0.002 | 1 |
| 32.0000 | 0 | 1 |

**Table S14. Coordinates of the Curve of sum scores of important features of ADHD in the second test set.**

| **Coordinates of the Curve** | | |
| --- | --- | --- |
| Test Result Variable(s): sum | | |
| Positive if Greater Than or Equal To^a^ | Sensitivity | Specificity |
| -1.0000 | 1 | 0 |
| .5000 | 0.964 | 0.282 |
| 1.5000 | 0.964 | 0.436 |
| 2.5000 | 0.945 | 0.645 |
| 3.5000 | 0.882 | 0.709 |
| 4.5000 | 0.855 | 0.782 |
| 5.5000 | 0.791 | 0.882 |
| 6.5000 | 0.718 | 0.936 |
| 7.5000 | 0.636 | 0.945 |
| 8.5000 | 0.518 | 0.973 |
| 9.5000 | 0.436 | 0.973 |
| 10.5000 | 0.345 | 1 |
| 11.5000 | 0.227 | 1 |
| 12.5000 | 0.136 | 1 |
| 13.5000 | 0.1 | 1 |
| 14.5000 | 0.073 | 1 |
| 15.5000 | 0.036 | 1 |
| 16.5000 | 0.009 | 1 |
| 18.0000 | 0 | 1 |

**Table S15. Coordinates of the Curve of sum scores of important features of anxiety problems in the second test set.**

| **Coordinates of the Curve** | | |
| --- | --- | --- |
| Test Result Variable(s): sum | | |
| Positive if Greater Than or Equal To^a^ | Sensitivity | Specificity |
| -1.0000 | 1 | 0 |
| .5000 | 0.971 | 0.196 |
| 1.5000 | 0.949 | 0.42 |
| 2.5000 | 0.906 | 0.529 |
| 3.5000 | 0.87 | 0.667 |
| 4.5000 | 0.797 | 0.717 |
| 5.5000 | 0.732 | 0.804 |
| 6.5000 | 0.659 | 0.855 |
| 7.5000 | 0.572 | 0.877 |
| 8.5000 | 0.478 | 0.913 |
| 9.5000 | 0.377 | 0.942 |
| 10.5000 | 0.304 | 0.957 |
| 11.5000 | 0.232 | 0.964 |
| 12.5000 | 0.188 | 0.971 |
| 13.5000 | 0.138 | 0.986 |
| 14.5000 | 0.101 | 0.993 |
| 15.5000 | 0.058 | 0.993 |
| 16.5000 | 0.043 | 0.993 |
| 17.5000 | 0.014 | 0.993 |
| 18.5000 | 0.007 | 1 |
| 20.0000 | 0 | 1 |

**Table S16. Coordinates of the Curve of sum scores of important features of conduct problems in the second test set.**

| **Coordinates of the Curve** | | |
| --- | --- | --- |
| Test Result Variable(s): sum | | |
| Positive if Greater Than or Equal To^a^ | Sensitivity | Specificity |
| -1.0000 | 1 | 0 |
| .5000 | 0.989 | 0.228 |
| 1.5000 | 0.902 | 0.391 |
| 2.5000 | 0.837 | 0.543 |
| 3.5000 | 0.728 | 0.717 |
| 4.5000 | 0.652 | 0.793 |
| 5.5000 | 0.533 | 0.837 |
| 6.5000 | 0.413 | 0.902 |
| 7.5000 | 0.304 | 0.946 |
| 8.5000 | 0.228 | 0.957 |
| 9.5000 | 0.13 | 0.967 |
| 10.5000 | 0.12 | 0.989 |
| 11.5000 | 0.076 | 1 |
| 12.5000 | 0.054 | 1 |
| 13.5000 | 0.043 | 1 |
| 14.5000 | 0.022 | 1 |
| 16.0000 | 0.011 | 1 |
| 18.0000 | 0 | 1 |

**Table S17. Coordinates of the Curve of sum scores of important features of depressive problems in the second test set.**

| **Coordinates of the Curve** | | |
| --- | --- | --- |
| Test Result Variable(s): sum | | |
| Positive if Greater Than or Equal To^a^ | Sensitivity | Specificity |
| -1.0000 | 1 | 0 |
| .5000 | 0.963 | 0.156 |
| 1.5000 | 0.944 | 0.356 |
| 2.5000 | 0.888 | 0.512 |
| 3.5000 | 0.863 | 0.662 |
| 4.5000 | 0.819 | 0.731 |
| 5.5000 | 0.719 | 0.819 |
| 6.5000 | 0.65 | 0.869 |
| 7.5000 | 0.556 | 0.912 |
| 8.5000 | 0.469 | 0.944 |
| 9.5000 | 0.431 | 0.963 |
| 10.5000 | 0.35 | 0.969 |
| 11.5000 | 0.263 | 0.969 |
| 12.5000 | 0.213 | 0.975 |
| 13.5000 | 0.156 | 0.994 |
| 14.5000 | 0.113 | 0.994 |
| 15.5000 | 0.081 | 1 |
| 16.5000 | 0.05 | 1 |
| 17.5000 | 0.038 | 1 |
| 19.0000 | 0.031 | 1 |
| 20.5000 | 0.025 | 1 |
| 21.5000 | 0.019 | 1 |
| 23.5000 | 0.006 | 1 |
| 26.0000 | 0 | 1 |

**Table S18. Coordinates of the Curve of sum scores of important features of oppositional defiant problems in the second test set..**

| **Coordinates of the Curve** | | |
| --- | --- | --- |
| Test Result Variable(s): sum | | |
| Positive if Greater Than or Equal To^a^ | Sensitivity | Specificity |
| -1.0000 | 1 | 0 |
| .5000 | 0.976 | 0.345 |
| 1.5000 | 0.964 | 0.536 |
| 2.5000 | 0.94 | 0.69 |
| 3.5000 | 0.857 | 0.762 |
| 4.5000 | 0.75 | 0.857 |
| 5.5000 | 0.643 | 0.881 |
| 6.5000 | 0.56 | 0.976 |
| 7.5000 | 0.429 | 0.988 |
| 8.5000 | 0.333 | 1 |
| 9.5000 | 0.274 | 1 |
| 10.5000 | 0.167 | 1 |
| 11.5000 | 0.095 | 1 |
| 12.5000 | 0.012 | 1 |
| 14.0000 | 0 | 1 |

**Table S19. Coordinates of the Curve of sum scores of important features of somatic problems in the second test set.**

| **Coordinates of the Curve** | | |
| --- | --- | --- |
| Test Result Variable(s): sum | | |
| Positive if Greater Than or Equal To^a^ | Sensitivity | Specificity |
| -1.0000 | 1 | 0 |
| .5000 | 0.97 | 0.152 |
| 1.5000 | 0.922 | 0.273 |
| 2.5000 | 0.892 | 0.403 |
| 3.5000 | 0.814 | 0.532 |
| 4.5000 | 0.745 | 0.654 |
| 5.5000 | 0.619 | 0.766 |
| 6.5000 | 0.567 | 0.831 |
| 7.5000 | 0.498 | 0.883 |
| 8.5000 | 0.411 | 0.939 |
| 9.5000 | 0.338 | 0.952 |
| 10.5000 | 0.286 | 0.965 |
| 11.5000 | 0.238 | 0.974 |
| 12.5000 | 0.182 | 0.983 |
| 13.5000 | 0.139 | 0.987 |
| 14.5000 | 0.104 | 0.991 |
| 15.5000 | 0.078 | 0.996 |
| 16.5000 | 0.065 | 0.996 |
| 17.5000 | 0.056 | 0.996 |
| 18.5000 | 0.043 | 0.996 |
| 19.5000 | 0.039 | 1 |
| 20.5000 | 0.03 | 1 |
| 22.0000 | 0.022 | 1 |
| 23.5000 | 0.013 | 1 |
| 24.5000 | 0.009 | 1 |
| 26.0000 | 0 | 1 |

**Table S20. AUC of other algorithm models including all features.**

| Logistic regression | | | | | | SVM | | | Multi-Layer Perceptron | | |
| --- | --- | --- | --- | --- | --- | --- | --- | --- | --- | --- | --- |
| ADHD | | | | | | | | | | | |
| AUC on the training set | | | | AUC on the test set | | AUC on the training set | | AUC on the test set | AUC on the training set | | AUC on the test set |
| Mean | | Std | |  | | Mean | Std |  | Mean | Std |  |
| 0.946 | | 0.034 | | 0.816 | | 0.946 | 0.031 | 0.843 | 0.930 | 0.045 | 0.718 |
| Anxiety Problem | | | | | | | | | | | |
| AUC on the training set | | | | AUC on the test set | | AUC on the training set | | AUC on the test set | AUC on the training set | | AUC on the test set |
| Mean | | Std | |  | | Mean | Std |  | Mean | Std |  |
| 0.916 | | 0.019 | | 0.832 | | 0.574 | 0.074 | 0.510 | 0.911 | 0.025 | 0.768 |
| Conduct Problem | | | | | | | | | | | |
| AUC on the training set | | | | AUC on the test set | | AUC on the training set | | AUC on the test set | AUC on the training set | | AUC on the test set |
| Mean | | Std | |  | | Mean | Std |  | Mean | Std |  |
| 0.917 | | 0.020 | | 0.840 | | 0.607 | 0.078 | 0.469 | 0.523 | 0.136 | 0.537 |
| Depressive Problem | | | | | | | | | | | |
| AUC on the training set | | | | AUC on the test set | | AUC on the training set | | AUC on the test set | AUC on the training set | | AUC on the test set |
| Mean | | Std | |  | | Mean | Std |  | Mean | Std |  |
| 0.900 | | 0.026 | | 0.830 | | 0.897 | 0.029 | 0.848 | 0.887 | 0.025 | 0.777 |
| Oppositional Defiant Problem | | | | | | | | | | | |
| AUC on the training set | | | AUC on the test set | | AUC on the training set | | | AUC on the test set | AUC on the training set | | AUC on the test set |
| Mean | Std | |  | | Mean | | Std |  | Mean | Std |  |
| 0.961 | 0.038 | | 0.767 | | 0.943 | | 0.027 | 0.813 | 0.946 | 0.047 | 0.741 |
| Somatic Problem | | | | | | | | | | | |
| AUC on the training set | | | | AUC on the test set | | AUC on the training set | | AUC on the test set | AUC on the training set | | AUC on the test set |
| Mean | | Std | |  | | Mean | Std |  | Mean | Std |  |
| 0.822 | | 0.006 | | 0.699 | | 0.530 | 0.27 | 0.521 | 0.764 | 0.023 | 0.616 |

**Table S21. AUC of other algorithm models only including CBCL items.**

| Logistic regression | | | | | | SVM | | | Multi-Layer Perceptron | | |
| --- | --- | --- | --- | --- | --- | --- | --- | --- | --- | --- | --- |
| ADHD | | | | | | | | | | | |
| AUC on the training set | | | | AUC on the test set | | AUC on the training set | | AUC on the test set | AUC on the training set | | AUC on the test set |
| Mean | | Std | |  | | Mean | Std |  | Mean | Std |  |
| 0.907 | | 0.038 | | 0.845 | | 0.903 | 0.040 | 0.841 | 0.881 | 0.048 | 0.829 |
| Anxiety Problem | | | | | | | | | | | |
| AUC on the training set | | | | AUC on the test set | | AUC on the training set | | AUC on the test set | AUC on the training set | | AUC on the test set |
| Mean | | Std | |  | | Mean | Std |  | Mean | Std |  |
| 0.865 | | 0.020 | | 0.882 | | 0.860 | 0.025 | 0.891 | 0.822 | 0.010 | 0.837 |
| Conduct Problem | | | | | | | | | | | |
| AUC on the training set | | | | AUC on the test set | | AUC on the training set | | AUC on the test set | AUC on the training set | | AUC on the test set |
| Mean | | Std | |  | | Mean | Std |  | Mean | Std |  |
| 0.913 | | 0.011 | | 0.843 | | 0.898 | 0.011 | 0.864 | 0.879 | 0.017 | 0.852 |
| Depressive Problem | | | | | | | | | | | |
| AUC on the training set | | | | AUC on the test set | | AUC on the training set | | AUC on the test set | AUC on the training set | | AUC on the test set |
| Mean | | Std | |  | | Mean | Std |  | Mean | Std |  |
| 0.881 | | 0.023 | | 0.873 | | 0.879 | 0.025 | 0.867 | 0.852 | 0.033 | 0.809 |
| Oppositional Defiant Problem | | | | | | | | | | | |
| AUC on the training set | | | AUC on the test set | | AUC on the training set | | | AUC on the test set | AUC on the training set | | AUC on the test set |
| Mean | Std | |  | | Mean | | Std |  | Mean | Std |  |
| 0.927 | 0.034 | | 0.881 | | 0.921 | | 0.044 | 0.864 | 0.946 | 0.047 | 0.839 |
| Somatic Problem | | | | | | | | | | | |
| AUC on the training set | | | | AUC on the test set | | AUC on the training set | | AUC on the test set | AUC on the training set | | AUC on the test set |
| Mean | | Std | |  | | Mean | Std |  | Mean | Std |  |
| 0.784 | | 0.040 | | 0.704 | | 0.771 | 0.042 | 0.692 | 0.720 | 0.032 | 0.666 |

**Table S22. All features we used in our models.**

| name | range1 | range2 |
| --- | --- | --- |
| MRI features | | |
| abcd_betnet02 | rsfmri_c_ngd_ad_ngd_ad | rsfmri_c_ngd_vs_ngd_vs |
| abcd_ddtidp101 | ddtidp_1 | ddtidp_981 |
| abcd_ddtidp201 | ddtidp_982 | ddtidp_1812 |
| abcd_ddtifp101 | ddtifp_1 | ddtifp_971 |
| abcd_ddtifp201 | ddtifp_972 | ddtifp_1812 |
| abcd_dmdtifp101 | dmdtifp1_1 | dmdtifp1_966 |
| abcd_dmdtifp202 | dmdtifp1_967 | dmdtifp1_1185 |
| abcd_drsip101 | dmri_rsi_meanmotion | dmri_rsirnigwc_cdx_meanrh |
| abcd_drsip201 | dmri_rsirnd_fib_fxrh | dmri_rsirndgwc_cdx_meanrh |
| abcd_drsip301 | dmri_rsirnt_fib_fxrh | dmri_rsirntgwc_cdx_meanrh |
| abcd_drsip701 | dmri_rsifni_fib_fxrh | dmri_rsifnigwc_cdx_meanrh |
| abcd_dti_p101 | dmri_dti_meanmotion | dmri_dtimdgm_cortdesikan_mean |
| abcd_dti_p201 | dmri_dtildgm_cdsn_bslh | dmri_dtitdgwc_cortdesikan_mean |
| abcd_midabwdp01 | midabwdp1 | midabwdp975 |
| abcd_midabwdp202 | midabwdp977 | midabwdp1481 |
| abcd_midasemdp101 | midasemdp1 | midasemdp950 |
| abcd_midasemdp202 | midasemdp951 | midasemdp1480 |
| abcd_midasemp102 | tfmri_mid_all_sem_tr | tfmri_ma_alvsl_sescs_valdcrh |
| abcd_midasemp202 | tfmri_ma_arvn_secdk_bstslh | tfmri_ma_alvsl_secdk_inarh |
| abcd_midr1bwdp101 | tfmrimr1_1 | tfmrimr1_971 |
| abcd_midr1bwdp202 | tfmrimr1_972 | tfmrimr1_1480 |
| abcd_midr1bwp102 | tfmri_mid_run1_beta_tr | tfmri_mr1_alvsl_bscs_vtdcrh |
| abcd_midr1bwp202 | tfmri_mr1_arvn_bcdk_bstslh | tfmri_mr1_alvsl_bcdk_ilarh |
| abcd_midr2semp102 | tfmri_mid_run2_sem_tr | tfmrimr2_300 |
| abcd_midr2semp202 | tfmrimr2_301 | tfmrimr2_980 |
| abcd_midsemp102 | tfmri_mid_run1_sem_tr | tfmri_mr1_alvsl_sscs_valdcrh |
| abcd_midsemp202 | tfmri_mr1_arvn_scdk_bstslh | tfmri_mr1_alvsl_scdk_isrh |
| abcd_mrirsfd01 | mrirsfd1 | mrirsfd148 |
| abcd_mrirstv02 | rsfmri_var_tr | rsfmri_var_scs_wmhypin |
| abcd_mrisdp102 | mrisdp_1 | mrisdp_604 |
| abcd_mrisdp202 | mrisdp_605 | mrisdp_1057 |
| abcd_mrisdp302 | mrisdp_1058 | mrisdp_1510 |
| abcd_smrip102 | smri_thick_cdk_banksstslh | smri_vol_scs_subcorticalgv |
| abcd_smrip202 | smri_t1ww02_cdk_banksstslh | smri_t1w_scs_ccat |
| abcd_smrip302 | smri_t2ww02_cdk_banksstslh | smri_t2w_scs_ccat |
| abcd_tfabwdp101 | tfabwdp_1 | tfabwdp_976 |
| abcd_tfabwdp201 | tfabwdp_977 | tfabwdp_1332 |
| abcd_tfnbr1semdp101 | tfnbr1semdp_1 | tfnbr1semdp_971 |
| abcd_tfnbr1semdp201 | tfnbr1semdp_972 | tfnbr1semdp_1332 |
| abcd_tfnbr2bwdp101 | tfnbr2bwdp_1 | tfnbr2bwdp_971 |
| abcd_tfnbr2bwdp201 | tfnbr2bwdp_972 | tfnbr2bwdp_1332 |
| abcd_tfnbr2dp101 | tfnbr2dp_1 | tfnbr2dp_971 |
| abcd_tfnbr2dp201 | tfnbr2dp_972 | tfnbr2dp_1332 |
| abcd_tfncr1bwdp101 | tfncr1bwdp_1 | tfncr1bwdp_971 |
| abcd_tfncr1bwdp201 | tfncr1bwdp_972 | tfncr1bwdp_1332 |
| abcd_tfsstabwdp101 | tfsstabwdp_1 | tfsstabwdp_916 |
| abcd_tfsstabwdp201 | tfsstabwdp_917 | tfsstabwdp_1036 |
| abcd_tfsstasemdp101 | tfsstasemdp_1 | tfsstasemdp_921 |
| abcd_tfsstasemdp201 | tfsstasemdp_922 | tfsstasemdp_1036 |
| abcd_tfsstr1bwdp101 | tfsstr1bwdp_1 | tfsstr1bwdp_921 |
| abcd_tfsstr1bwdp201 | tfsstr1bwdp_922 | tfsstr1bwdp_1036 |
| abcd_tfsstr1semdp101 | tfsstr1semdp_1 | tfsstr1semdp_921 |
| abcd_tfsstr1semdp201 | tfsstr1semdp_922 | tfsstr1semdp_1036 |
| abcd_tfsstr2bwdp101 | tfsstr2bwdp_1 | tfsstr2bwdp_921 |
| abcd_tfsstr2bwdp201 | tfsstr2bwdp_922 | tfsstr2bwdp_1036 |
| abcd_tfsstr2semdp101 | tfsstr2semdp_1 | tfsstr2semdp_921 |
| abcd_tfsstr2semdp201 | tfsstr2semdp_922 | tfsstr2semdp_1036 |
| abcd_tmidr1semdp101 | tmidr1semdp_1 | tmidr1semdp_971 |
| abcd_tmidr1semdp202 | tmidr1semdp_972 | tfmrimr1_1631 |
| abcd_tnbasemdp101 | tnbasemdp_1 | tnbasemdp_971 |
| abcd_tnbasemdp201 | tnbasemdp_972 | tnbasemdp_1332 |
| abcd_tr2bwdp01 | tr2bwdp_1 | tr2bwdp_971 |
| abcd_tr2bwdp202 | tr2bwdp_972 | tr2bwdp_1480 |
| abcd_tr2semdp101 | tr2semdp_1 | tr2semdp_975 |
| abcd_tr2semdp201 | tr2semdp_976 | tr2semdp_1480 |
| midaparc03 | tfmri_mid_all_b_tr | tfmri_ma_alvsl_b_scs_vtdcrh |
| midaparcp203 | tfmri_ma_arvn_b_cds_bkslh | tfmri_ma_alvsl_b_cds_insularh |
| midr2bwp102 | tfmri_mr2_beta_tr | tfmri_mr2_alvsl_bscs_vtraldcrh |
| midr2bwp202 | tfmri_mr2_arvn_bcdk_bktslh | tfmri_mr2_alvsl_bcdk_inarh |
| mrirscor02 | rsfmri_cor_ngd_scs_tr | rsfmri_cor_ngd_vs_scs_vtdcrh |
| mrisst02 | tfmri_sa_beta_tr | tfmri_saigvis_bcdk_isarh |
| mrisstr1bw01 | tfmri_sstr1_beta_tr | tfmri_sstr1_686 |
| mrisstr1sem01 | tfmri_sst_r1_sem_tr | tfmri_sst_r1sem_686 |
| mrisstr2bw01 | tfmri_sstr2_beta_tr | tfmri_sstr2_686 |
| mrisstr2bwsem01 | tfmri_sst_r2_sem_tr | tfmri_sst_r2sem_686 |
| mrisstsem01 | tfmri_sst_all_sem_tr | tfmri_sst_all_686 |
| nback_bwroi02 | tfmri_nback_all_beta_tr | tfmri_nback_all_882 |
| nbackallsem01 | tfmri_nback_all_sem_tr | tfmrinbackallsem_882 |
| nbackr101 | tfmri_nback_run1_beta_tr | tfmri_nback_r1_882 |
| nbackr1sem01 | tfmri_nback_r1_sem_tr | tfmri_nback_r1sem_882 |
| nbackr201 | tfmri_nback_r2_beta_tr | tfmri_nback_r2_882 |
| nbackr2sem01 | tfmri_nbr2_sem_tr | tfmrinbr2pfvnfscdkinsularh |
| Clinical Features | | |
| abcd_lpds01 | demo_prnt_race_acs_p__10 | demo_prnt_race_acs_p__999 |
|  | demo_prnt_race_a_v2_l___10 | demo_prnt_race_a_v2_l___99 |
| abcd_pnsc01 | neighborhood1r_p | neighborhood3r_p |
| abcd_nsc01 | neighborhood_crime_y | neighborhood_crime_y |
| abcd_bisbas01 | bisbas1_y | bisbas5r_y |
| abcd_fes01 | fes_youth_q1 | fes_youth_q9 |
| fes02 | fam_enviro1_p | fam_enviro_select_language___1 |
| srpf01 | school_2_y | school_17_y |
| abcd_stq01 | screen1_wkdy_y | screen14_y |
| abcd_stq01 | screentime_smq_use___7 | screentime_smq_use___6 |
| pmq01 | parent_monitor_q1_y | parent_monitor_q5_y |
| abcd_ehis01 | ehi1b | ehi4b |
|  | ehi_y_ss_scoreb | ehi_y_ss_scoreb |
| abcd_tbss01 | nihtbx_picvocab_uncorrected | nihtbx_picvocab_agecorrected |
|  | nihtbx_flanker_uncorrected | nihtbx_flanker_agecorrected |
|  | nihtbx_list_uncorrected | nihtbx_list_agecorrected |
|  | nihtbx_cardsort_uncorrected | nihtbx_cardsort_agecorrected |
|  | nihtbx_pattern_uncorrected | nihtbx_pattern_agecorrected |
|  | nihtbx_picture_uncorrected | nihtbx_picture_agecorrected |
|  | nihtbx_reading_uncorrected | nihtbx_reading_agecorrected |
|  | nihtbx_fluidcomp_uncorrected | nihtbx_fluidcomp_agecorrected |
|  | nihtbx_cryst_uncorrected | nihtbx_cryst_agecorrected |
|  | nihtbx_totalcomp_uncorrected | nihtbx_totalcomp_agecorrected |
|  | nihtbx_picvocab_theta | nihtbx_picvocab_itmcnt |
|  | nihtbx_picvocab_fc | nihtbx_reading_itmcnt |
|  | nihtbx_reading_fc | nihtbx_reading_fc |
|  | nihtbx_fluidcomp_fc | nihtbx_fluidcomp_fc |
|  | nihtbx_cryst_fc | nihtbx_cryst_fc |
|  | nihtbx_totalcomp_fc | nihtbx_totalcomp_fc |
| abcd_sds01 | sleepdisturb1_p | sleepdisturb26_p |
| fhxp102 | famhx_1 | famhx_1a_p |
|  | famhx_1b_p | famhx_1b_p |
|  | famhx_2a_p | famhx_2a_p |
|  | famhx_2b_p | famhx_2b_p |
|  | fhx_3a_p | fhx_3a_p |
|  | fhx_3b_p | fhx_3b_p |
|  | fhx_3ha_p | fhx_3ha_p |
|  | fhx_3hb_p | fhx_3hb_p |
|  | famhx_4_p | q5m_full_sib_same2_drugs___6 |
| dibf01 | kbi_p_c_live_full_time | kbi_p_c_best_friend |
|  | kbi_p_c_reg_friend_group | kbi_p_c_reg_friend_group |
|  | kbi_p_c_bully | kbi_p_c_gay |
|  | kbi_p_c_trans | kbi_p_c_trans |
|  | kbi_ss_c_substance_abuse_p | kbi_ss_c_substance_abuse_p |
| pdem02 | demo_prnt_ed_v2 | demo_prnt_ed_v2 |
| CBCL | | |
| abcd_cbcl01 | cbcl_q01_p | cbcl_q112_p |
